# Cannabinoids-Promising Antimicrobial Drugs or Intoxicants with Benefits?

**DOI:** 10.3390/antibiotics9060297

**Published:** 2020-06-02

**Authors:** Philipp Klahn

**Affiliations:** Institute of Organic Chemistry, Technische Universität Braunschweig, Hagenring 30, D-38106 Braunschweig, Germany; p.klahn@tu-braunschweig.de

**Keywords:** cannabinoids, antimicrobial drug, microbial resistance, methicillin-resistant *Staphylococcus aureus* (MRSA), broad-spectrum antibiotics, antibiofilm activity, persister cell killing

## Abstract

Novel antimicrobial drugs are urgently needed to counteract the increasing occurrence of bacterial resistance. Extracts of *Cannabis sativa* have been used for the treatment of several diseases since ancient times. However, its phytocannabinoid constituents are predominantly associated with psychotropic effects and medical applications far beyond the treatment of infections. It has been demonstrated that several cannabinoids show potent antimicrobial activity against primarily Gram-positive bacteria including methicillin-resistant *Staphylococcus aureus* (MRSA). As first in vivo efficacy has been demonstrated recently, it is time to discuss whether cannabinoids are promising antimicrobial drug candidates or overhyped intoxicants with benefits.

## 1. Introduction

The worldwide spread of bacterial resistance against market antibiotics has been identified as one of the major threats to public health by scientists and healthcare authorities [1,2,3,4]. Thus, new antibacterial strategies and antibacterial compounds are urgently needed to counteract the increasing occurrence of antibiotic-resistant and, especially, multidrug-resistant (MDR) pathogens, to keep the live-saving advantages toward bacterial pathogens [5,6]. In 2017, the World Health Organization (WHO) emphasized the crucial need for antimicrobial drug development against a group of 13 different genii, families, and specific species of pathogenic bacteria by categorizing them into critical, high, and medium priority groups, according to their prevalence of resistance, clinical relevance, contributions to global morbidity and mortality, and their economic burden, for global healthcare systems to guide antimicrobial research and development [7,8]. The vast majority of these prioritized bacterial pathogens are drug-resistant Gram-negative bacteria. The development of new antimicrobial drugs against Gram-negative bacteria is particularly challenging due to the general structure of the Gram-negative cell envelope, which leads to a very effective permeation barrier rendering a majority of drugs with antibacterial activity against Gram-positive pathogens inactive against Gram-negative ones.

However, antimicrobial resistances are also increasing in Gram-positive bacteria and, among the high priority pathogens, the Gram-positive pathogen *Staphylococcus aureus* is the leading cause of both healthcare and community-associated infections worldwide, and a major cause for morbidity as well as mortality, especially taking into account the emergence and rapid spread of Methicillin-resistant *Staphylococcus aureus* (MRSA). These bacteria can cause serious infections of the skin, surgical wounds, the bloodstream, the lungs, or the urinary tract. Furthermore, their distinct ability to form biofilms on tissue [9], in wounds [10,11], and on implants [12] can lead to severe chronic biofilm-associated infections [13,14].

Natural products have always been a rich source for the identification of antimicrobial drug candidates; thus, researchers have started to reinvestigate long known natural product-based drugs in order to provide solutions to the current antibiotic crisis [15,16,17,18,19,20,21,22]. Interestingly, different phytocannabinoids, natural product constituents of the extracts of the plant *Cannabis sativa*, which have so far mainly been associated with intoxication effects upon recreational usage and medical applications far beyond the treatment of infections, have been reported to show antibacterial activity against several Gram-positive bacterial pathogens, including MRSA [23,24,25,26,27,28,29].

This review article aims to give a concise overview on structure, biosynthesis, synthesis, and biological activities of natural phytocannabinoids and synthetic analogues to review and discuss early and recent investigations on the antibacterial properties of phytocannabinoids.

## 2. Structures, Abundance, and Biosynthetic Origin of Natural Cannabinoids from *Cannabis sativa*

**Figure 1:**
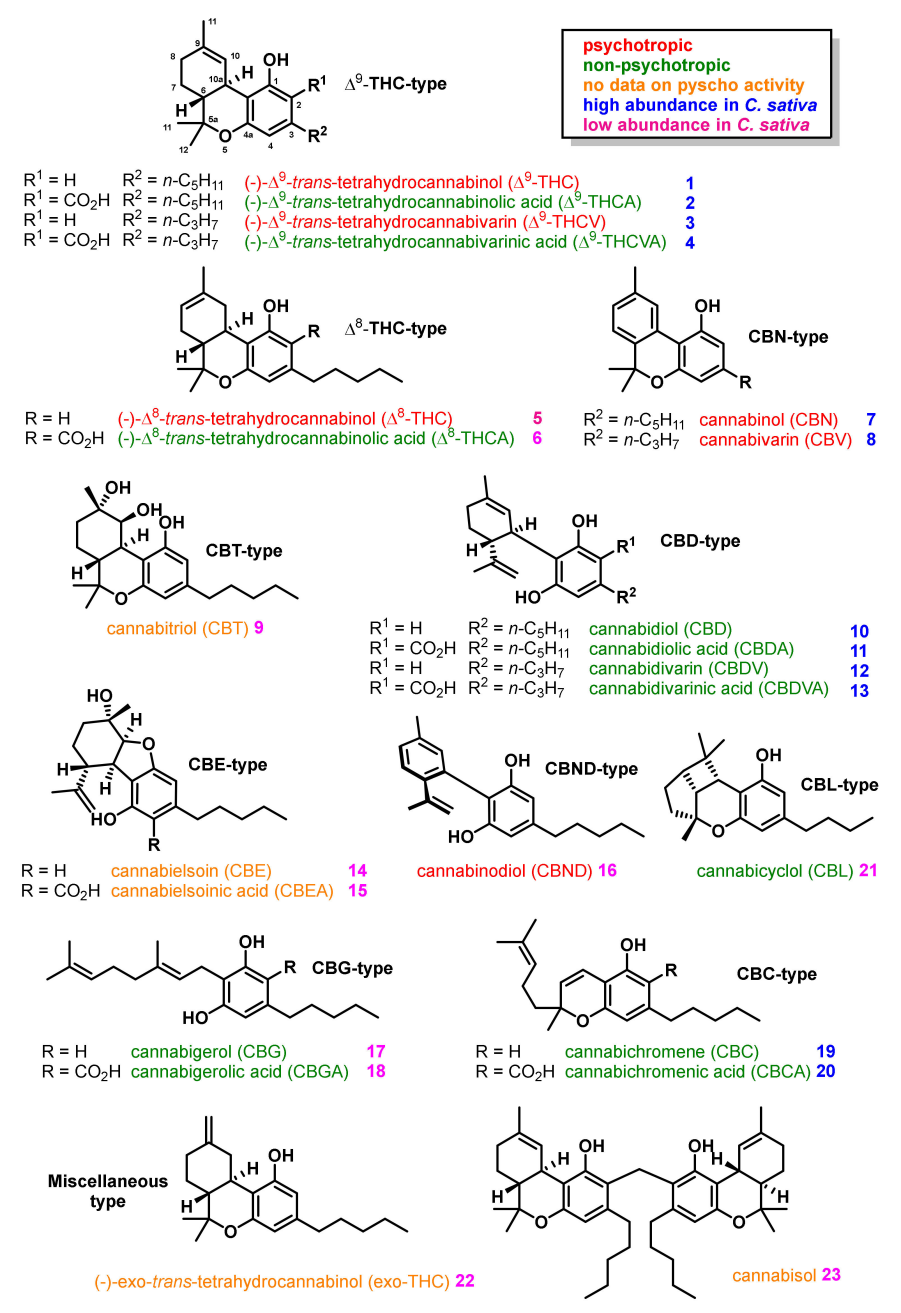
Overview on structures of general types of phytocannabinoids from *Cannabis sativa*, their psychotropic activities, and abundance.

Phytocannabinoids naturally occurring in *Cannabis sativa* typically comprise either a C21 or C22 terpenophenolic skeleton differing in their state of cyclization and oxidation pattern. To date, over 120 natural cannabinoids are known, which can be classified into 11 types of general structural frameworks, as outlined in Figure 1 [30,31,32]. The class of Δ^9^-THC-type cannabinoids [32] share a tricyclic 6a,7,8,10a-tetrahydro-6*H*-benzo[c]chromen-1-ol core structure, its major representatives (−)-Δ^9^-*trans*-tetrahydrocannabinol (Δ^9^-THC) (**1**) and (−)-Δ^9^-*trans*-tetrahydrocannabinolic acid (Δ^9^-THCA) (**21**) belong to the cannabinoids with high abundance in *Cannabis sativa* [33,34].

The class of Δ^8^-THC-type cannabinoids [32] contain isomers of Δ^9^-THC-type cannabinoids exhibiting a similar 6a,7,10,10a-tetrahydro-6*H*-benzo[c]chromen-1-ol structure with a shifted double bond. As a major representative of this type, (−)-Δ^8^-*trans*-Tetrahydrocannabinol (Δ^8^-THC) (**5**) is regarded as an artefact from the isomerization from the thermodynamically less stable double bond isomer Δ^9^-THC (**1**) [35,36,37,38] and its concentration in the plants is typically minuscule [33].

The cannabinol (CBN)-type cannabinoids [32] share a common 6*H*-benzo[c]chromen-1-ol core structure with an oxidized aromatic upper ring. The major representative of this class, cannabinol (CBN) (**7**), is a relatively minor constituent in fresh material of *Cannabis sativa* [31,33]. However, CBN (**7**) content increases in plant material upon storage by oxidation Δ^9^-THC (**1**) in the presence of oxygen [39]. Processed cannabis products, such as traditionally made hashish for recreational usage or cannabis oils generated by steam distillation, contain higher amounts of the thermodynamically more stable cannabinoids Δ^8^-THC (**5**) and CBN (**7**). Further, minor constituents from *Cannabis sativa* are the CBT-type cannabinoids [32] such as cannabitriol (CBT) (**9**) exhibiting a vicinal 9,10-*trans*-diol in the upper ring as structural feature clearly distinguishable from the Δ^9^-THC-type cannabinoids sharing the same carbon framework.

The CBD-type cannabinoids [32] such as cannabidiol (CBD) (**10**) display a tetrahydro-[1,1′-biphenyl]-2,6-diol framework with high abundance in *Cannabis sativa* and can make up to 40% of its dried extracts [40]. However, CBD (**10**) is intrinsically instable and cyclizes under acidic conditions to Δ^9^-THC (**1**) [36]. This cyclization as well as further oxidation of Δ^9^-THC (**1**) to CBN (**7**) also partially takes place during pyrolysis when cannabis is smoked [41] The CBE-type cannabinoids [32] such as cannabielsoin (CBE) (**14**) comprise a 5a,6,7,8,9,9a-hexahydrodibenzo[b,d]furan-1,6-diol framework, and have been demonstrated to be products of a oxidative photocyclization of CBD-type cannabinoids [42,43]. CBND-type cannabinoids [44] such as cannabinodiol (CBND) (**16**) show a [1,1′-biphenyl]-2,6-diol framework derived from CBD-type cannabinoids by aromatization of the upper ring and found in cannabis at low concentrations [45].

CBG-type cannabinoids [32], such as cannabigerol (CBG) (**17**), exhibit a non-cyclized framework with a benzene-1,3-diol core structure, and are minor constituents in *Cannabis sativa* as they are normally converted into Δ^9^-THC-type cannabinoids during plant growth, leaving only about 1% CBG (**7**) in dried extracts of the plant [46]. CBC-type cannabinoids [32] are a class showing a 2H-chromen-5-ol core structure, and its major representative cannabichromene (CBC) (**19**) is one of the most abundant cannabinoids in *Cannabis sativa* [47]. The exposure of (CBC) (**19**) to sunlight leads to a [2 + 2]-photocycloaddition forming cannabicyclol (CBL) (**21**) [48]. Finally, there is a very diverse group of miscellaneous-type cannabinoids, which can be isolated from *Cannabis sativa* in small amounts such as (−)-exo-*trans*-tetrahydrocannabinol (exo-THC) (**22**) or the dimeric cannabisol (**23**).

Furthermore, less abundant in *Cannabis sativa* are phytocannabinoids presenting a C19 and C22 terpenophenolic skeleton, such as cannabidivarin (CBDV) (12) or (−)-Δ^9^-trans-tetrahydrocannabivarin (Δ^9^--THCV) (**3**) (Figure 1), which is regarded to different biosynthetic precursors [49,50,51].

**Scheme 1:**
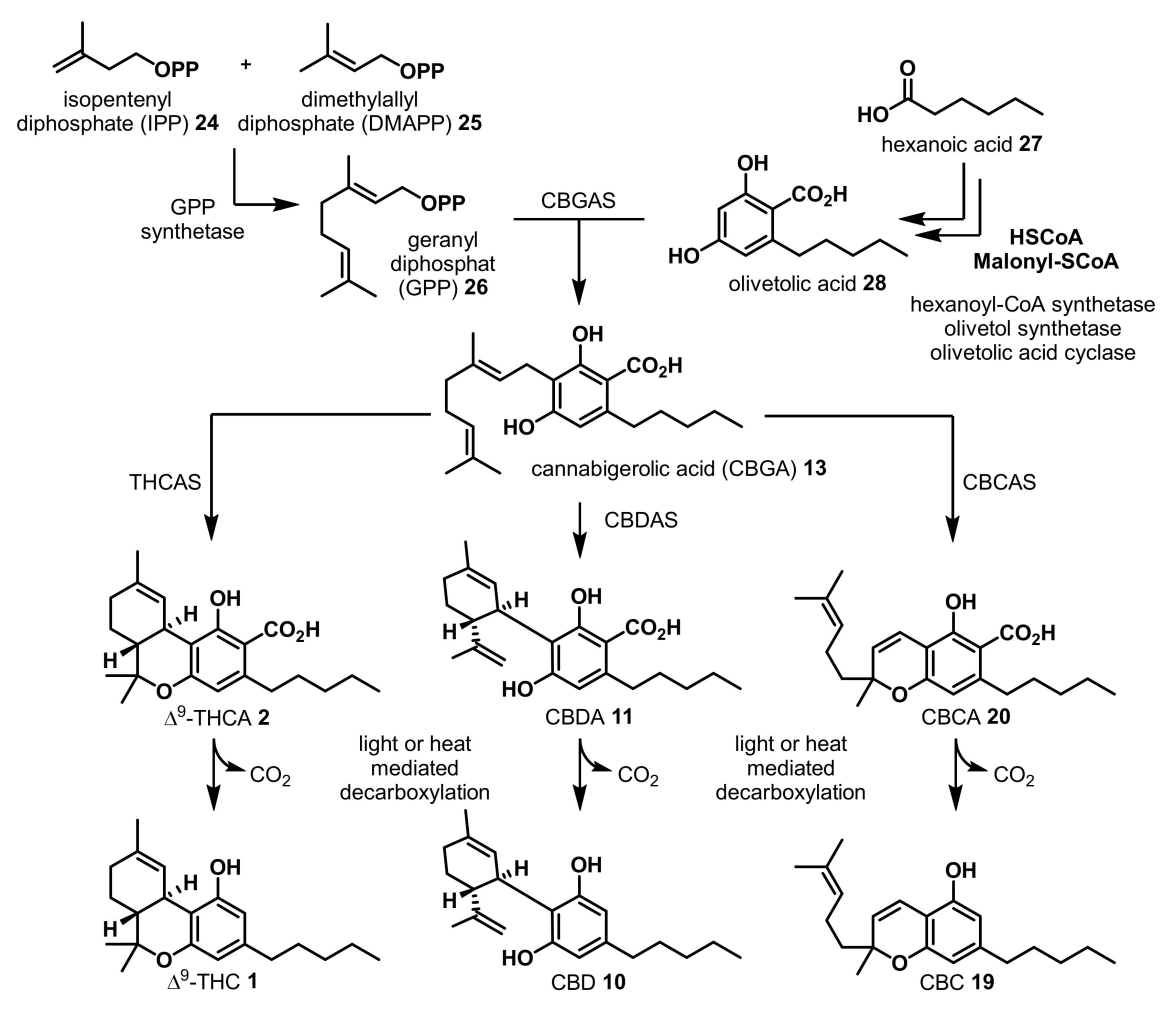
Overview on biosynthetic origin of the different phytocannabinoid structures from *Cannabis sativa*.

The terpenophenolic skeleton on phytocannabinoids biosynthetically originates from the monoterpene precursor geranyl diphosphate (GPP) (**26**) and olivetolic acid (**28**), which is produced by a polyketide synthetase (PKS) [52] and the olivetolic acid cyclase [53] (Scheme 1). The cannabigerolic acid synthetase (CBGAS) catalyzes the prenyl transfer via an electrophilic aromatic substitution leading to cannabigerolic acid (CBGA) (**13**) [54,55] CBGA (**13**) can further be oxidatively cyclized by the Δ^9^-tetrahydrocannabinolic acid synthetase (THCAS) to form (-)-Δ^9^-*trans*-tetrahydrocannabinolic acid (Δ^9^-THCA) (**2**) [56].

In contrast, cannabidiolic acid synthetase (CBDAS) catalyzes the formation of cannabidiolic acid (CBDA) (**11**) through an oxidative cyclization of CBGA (**13**) [57]. Cannabichromenic acid (CBCA) (**20**) is generated via oxidative cyclization of CBGA (**13**), catalyzed by the cannabichromenic acid synthetase (CBCAS) [58,59]. Sunlight or heat mediated spontaneous decarboxylation of Δ^9^-THCA (**2**), CBDA (**11**), or CBCA (**20**) then leads to the formation of the corresponding Δ^9^-THC (**1**), CBD (**10**), or CBC (**19**), respectively [49,50,51]. This decarboxylation reaction also takes place to a certain extent during baking or smoking of cannabis material [60].

## 3. Bioactivities and Medical Uses of Phytocannabinoids from *Cannabis Sativa* and Synthetic Analogues

Historically, different plant material, oils, and extracts of *Cannabis sativa* have been utilized for recreational, spiritual, and medical purposes since ancient times [61,62,63,64].

The strong psychotropic activity observed when marihuana (dried resinous flowers and proximal little leaves of *Cannabis sativa*) or hashish (dried and compressed resinous extracts of *Cannabis sativa*) are smoked, vaporized, or consumed orally; having been processed into pastries is related to the interaction of contained psychotropic cannabinoids as partial agonist with human endocannabinoid receptors CBR1 [65,66,67,68,69].

**Figure 2:**
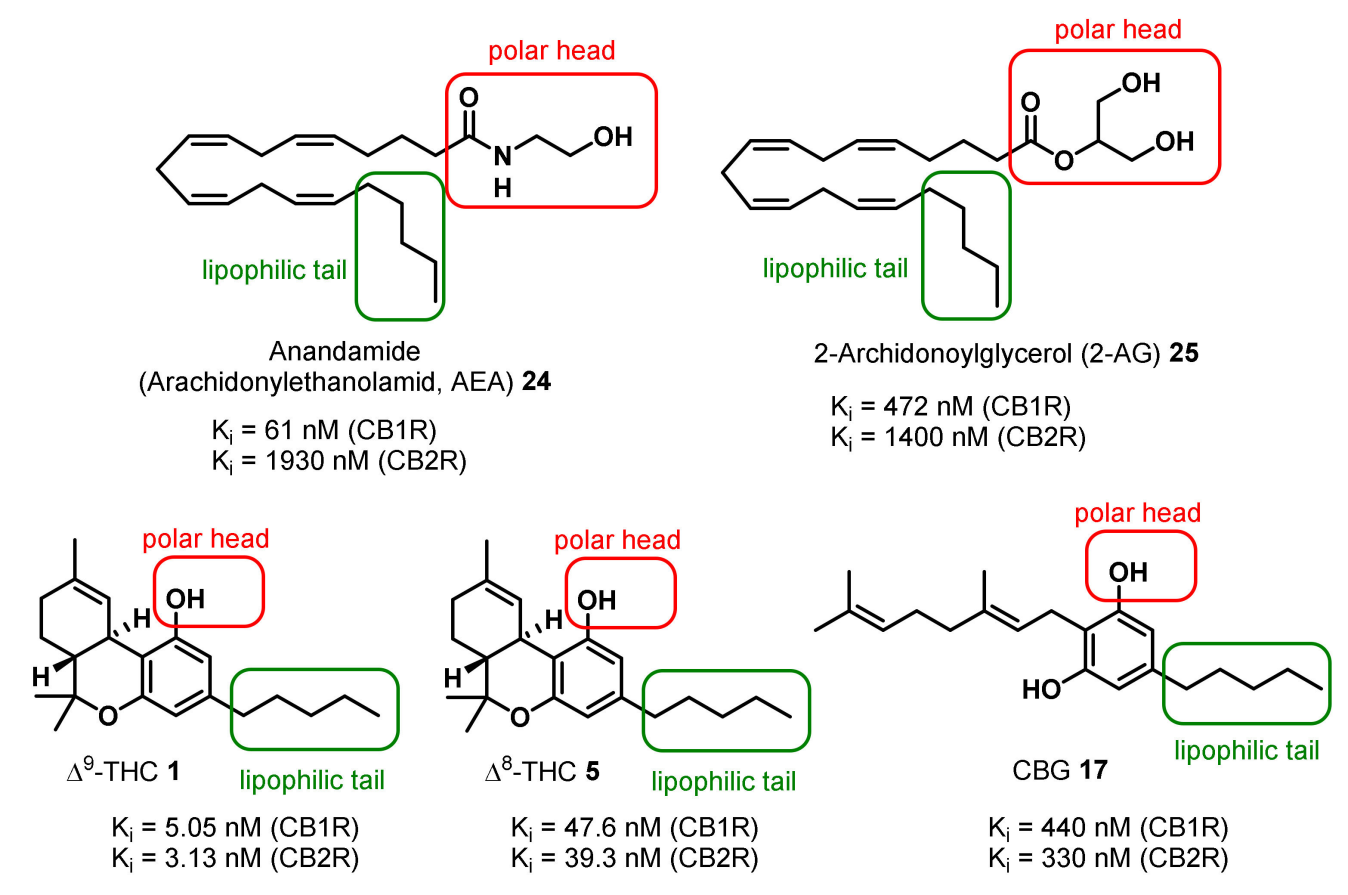
Structures of the endocannabinoids anandamide and 2-archidonoylglycerol and comparison of their binding affinities to cannabinoid receptors (CB1R and CB2R) to selected phytocannabinoids [74,75].

The human endocannabinoids systems (ECS) is a ubiquitous and complex system of endogenously produced endocannabinoids, such as anandamide (AEA) (24) and 2-archidonoylglycerol (2-AG) (25) (Figure 2), their G-protein coupled cannabinoid receptors (CBRs), enzymes for production and degradation of endogenous cannabinoids and proteins, which regulate the uptake and transport of endocannabinoids. The ECS is involved in almost all aspects of mammalian physiology and pathology [65,66,70,71]. The two main subclasses of CBRs are CB1R, which predominates in the central nervous system, and the principally peripheral cannabinoid receptor CB2R [65,66,70,72,73].

**Figure 3:**
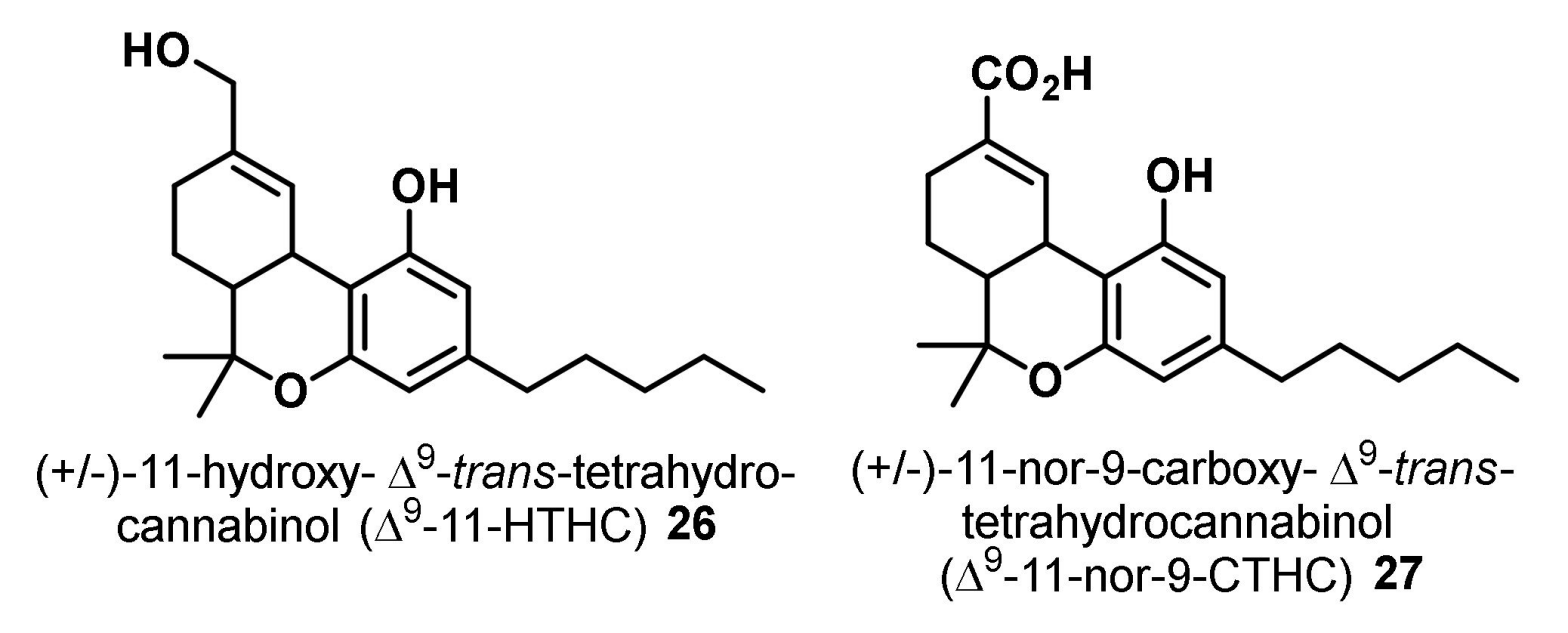
Structures of the Δ^9^-THC metabolites Δ^9^-11-HTHC and Δ^9^-11-nor-9-CTHC.

Δ^9^-THC (**1**) is the primary psychotropic compound in *Cannabis sativa* [33,34], displaying a binding affinity to CB1R in the low nanomolar range acting as a partial agonist (Figure 2). In addition, 11-hydroxy-Δ^9^-THC (**26**) (Figure 3), a primary metabolite in human produced upon cytochrome-P450 oxidation of Δ^9^-THC (**1**) has been found to have psychotropic activity. However, the follow up metabolite 11-nor-9-carboxy-Δ^9^-THC (**27**) (Figure 3) generated by further oxidation is non-psychotropic. Further psychotropic cannabinoids are Δ^8^-THC (**5**), (−)-Δ^9^-*trans*-tetrahydrocannabivarin (Δ^9^-THCV) (**3**), (CBN) (**7**), Cannabivarin (CBV) (**8**), and CBND (**16**) (Red highlighting in Figure 1) [33,34,45].

In contrast, all biosynthetic acid precursor cannabinoids, such as Δ^9^-THCA (**2**), Δ^9^-THCVA (**4**) or Δ^8^-THCA (**6**) do not possess psychotropic activity, similarly all CBC-type, CBD-type, CBG-type, CBL-type, and presumably also CBE-type cannabinoids, belong to the non-psychotropic representatives (green highlighting in Figure 1 and Figure 2) [33,34,45].

Beyond the psychotropic activity of cannabinoids, which has been limiting medical applications for a long time, an overwhelmingly broad variety of potentially beneficial effects of certain psychotropic and non-psychotropic cannabinoids, such as appetite stimulation, reduction of the antrum motility, anorectic, analgesic, antiglaucoma, anti-nausea, antiemetic, anti-inflammatory, antianxiety, antipsychotic, antidiabetic, neuroprotective, antipsoriatic, anti-ischemic, vasorelaxant, antiepileptic, antispasmolytic, bone-stimulant, as well as antitumor properties, have been reported and intensively reviewed elsewhere [33,62,76,77,78,79,80,81,82,83,84,85,86]. In most cases these effects are the consequence of a complex pharmacology with the same cannabinoid acting concentration dependent in a different manner at multiple molecular targets within the endocannabinoid and the expanded endocannabinoid system [71], or even targets outside of it [67,68].

For several of the phytocannabinoids, especially the minor constituents of *Cannabis sativa,* the overall pharmacological profile is still incomplete or there are no pharmacological data available. In general, more investigations are necessary to understand their complex biophysiological activities.

Medical cannabis, as well as several *Cannabis sativa* derived products, which have been approved by the national drug approval authorities, contain mostly either Δ^9^-THC (**1**), CBD (**10**), or a mixture of both cannabinoids. They are prescribed in several Western countries to allay symptoms, such as neuropathic pain, nausea, vomiting, muscle spastics, and weight loss within treatment of severe diseases, such as multiple sclerosis, epilepsy, AIDS, or cancer [33,62,83,87,88].

**Figure 4:**
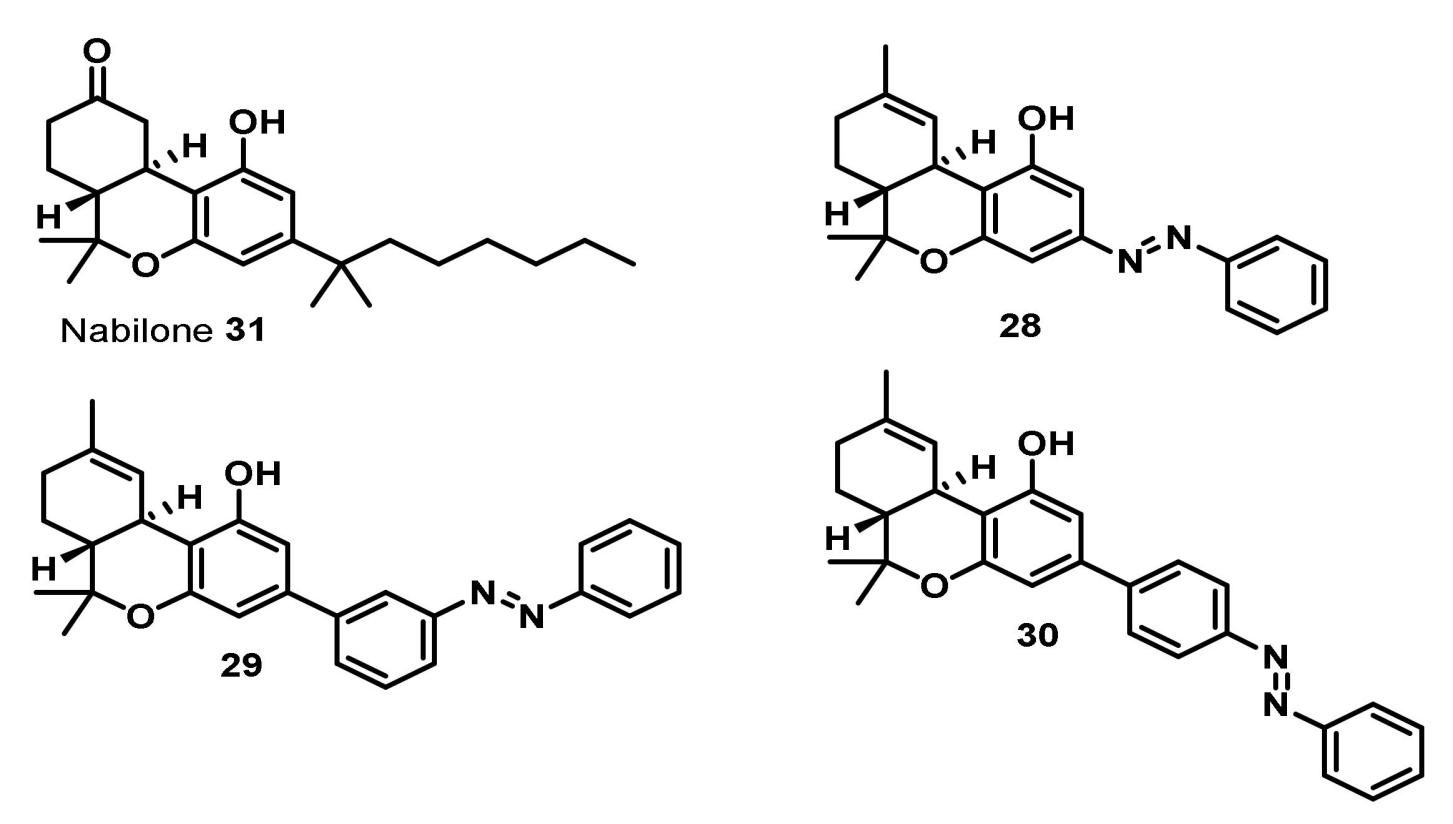
Structure of Nabilone, an approved fully synthetic Δ^9^-THC analogue and photo-switchable azo-THC analogues.

The broad variety of therapeutic effects and interesting bioactivities of phytocannabinoids has also stimulated their chemical total synthesis, bioengineered synthesis, as well as the synthesis of numerous analogues [75] with modified pharmacological profiles, which has recently been comprehensively reviewed [50,89,90,91]. A particular highlight is the synthesis and in vitro evaluation of potent photo-switchable Δ^9^-THC-analogues, which were reported by a consortium of the academic research groups of Trauner, Carreira, Frank, and their co-workers in 2017. These analogues (**28**–**31**, Figure 4) contained azobenzene moieties as lipophilic side chains, which allow optical control of CB1R signaling [92].

To date, fully synthetic, but naturally occurring ultra-pure cannabinoids, such as Dronabinol (synthetic Δ^9^-THC (**1**)) and even the fully synthetic Δ^9^-THC-analogue Nabilone (**31**) (Figure 4) bearing a modified lipophilic side chain and a ketone moiety in the upper ring are approved for different indications [87].

The synthetic exploration of the structure-activity relationships has also produced several cannabinoid mimetics with tremendously increased psychotropic activity, which entered the market of legally available designer drugs sold as herbal bath additives, leading to severe acute psychotropic and physical effects for consumers [93,94], and several cases of reported deaths upon acute organ failure [95]. Furthermore, in recent years cannabis or cannabinoids have become some sort of trend medication for several diseases strongly hyped by a growing cannabis lobby without significant medical data evidence. In 2019, US authorities for the Food and Drug Administration (FDA) published two consecutive warnings for misuse of cannabidiol products with unsubstantiated claims by producers [96,97].

## 4. Antimicrobial Activities of Phytocannabinoids from *Cannabis Sativa*

The application of plant material or seeds of *Cannabis sativa* for the preparation of early antibiotic treatments of different infections have already been reported from folk medicine at the end of the 19 th century, as described in early review articles by Kabelik [98,99].

First systematical evidence for the antimicrobial activity of constituents of *Cannabis sativa* was given by the Dissertation Krejci in 1950 [100]. His comprehensive bacteriological in vitro investigations on the antibacterial effects of extracts of *Cannabis sativa* on different bacteria were later published in 1958 [101]. Krejci reported a good activity of the cannabis extracts against the growth of Gram-positive bacteria, while Gram-negative bacteria were not affected. Furthermore, he prepared a salve containing 2% extracts and demonstrated its therapeutic efficacy in the treatment of skin infections with *Staphylococci* [101]. Independently, Drobotko et al. described the occurrence of antibacterial activity in extracts from hemp in 1951 [102], while Ferenczy et al. reported the finding of the occurrence of antibacterial activity in extracts obtained from seeds of hemp in 1956 [103,104]. Ferenczy et al. demonstrated the potency of the antimicrobial activity using dilutions of their extracts and observed that their activity was pH-dependent [104].

Since these early findings, numerous of reports on the antimicrobial activity of different extracts prepared from *Cannabis sativa* on Gram-positive, Gram-negative bacteria, and different fungi [105,106,107,108,109,110,111,112,113,114,115,116,117,118,119,120,121,122,123,124,125,126,127,128,129], which have also been partially reviewed elsewhere [98,130,131].

The additional scientific knowledge from these studies was rather limited, as in most cases, complex mixtures with undetermined composition or partially determined mixtures of over 60 compounds [112] have been utilized for the evaluation of the antimicrobial activity, thus, clear compound specific pharmacological conclusions are impossible. Considering the variety of natural products from different classes, which have been isolated from *Cannabis sativa* [132,133,134] and the complex pharmacology of phytocannabinoids mentioned above, studies on isolated single cannabinoids are required to fully understand their antimicrobial potential.

**Figure 5:**
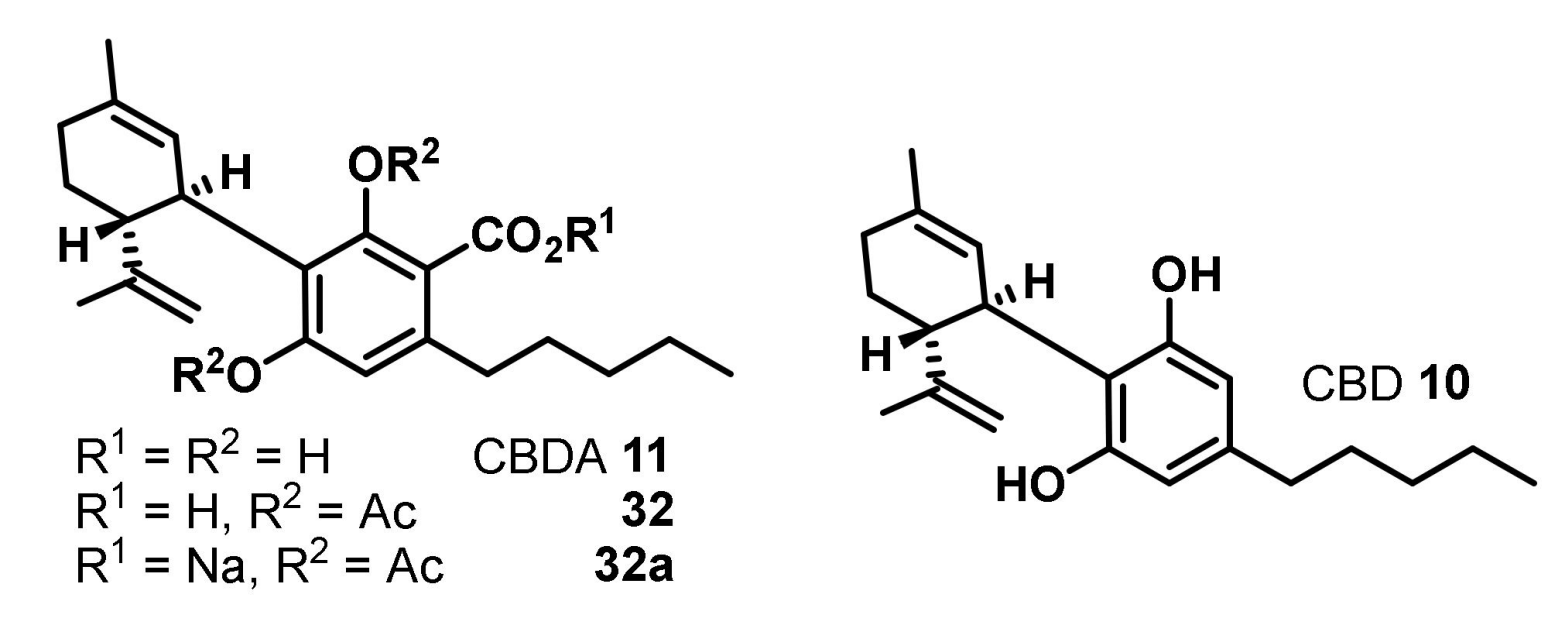
Structure of CBD (**10**), CBDA (**11**) and compounds **32** and **32a**.

The first early study on the antimicrobial activity of specific, isolated cannabinoids from *Cannabis sativa* were reported by Schultz and Haffner in 1958 [27]. They isolated cannabidiolic acid (CBDA) (**11**) and compared its antibacterial activity against *S. aureus*, *Bacillus subtilis,* and *Escherichia coli* to cannabidiol (CBD) (**10**), the corresponding diacetate (**31**) as well as its sodium salt (**32**) (Figure 5).

While CBD (**10**) and CBDA (**11**) show a potent antibacterial activity against the Gram-positive bacteria *S. aureus* and *B. subtilis*, an activity against the Gram-negative *E. coli* was not observed. As the poor water solubility of the compounds was limiting their investigations, the diacetate **32** and its sodium salt **32a** were generated, which showed significantly increased water solubility. Nonetheless, these derivatives were inactive against all tested bacteria [27].

A second study comparing the antibiotic activity of CBD (**10**), CBDA (**11**), and the acetylated cannabidiolic acid **32** was published by Krejci et al. in 1959 [28]. In contrast to the investigations of Schultz and Haffner [27], **32** displayed still potent antibacterial activity, although decreased compared to CBD (**10**) and CBDA (**11**) [28].

In 1965, Melchoulam et al. mentioned the antibacterial activity of CBG (**17**) and CBGA (**18**) against Gram-positive bacteria in a short side note of their publication on the isolation and structure determination of natural cannabinoids. However, neither information on bacterial strains nor specific MIC (Minimal Inhibitory Concentration) data were given [135].

In 1976, Klingeren and Ham published a study on the antibacterial activity of Δ^9^-THC (**1**) and CBD (**10**), in which both cannabinoids were screened for their antibacterial activity against the Gram-positive bacteria *S. aureus, Streptococcus pyogenes, Streptococcus milleri* and *Enterococcus faecalis* and the Gram-negative pathogens *E. coli, Salmonella typhi* and *Proteus vulgaris* [26]. Both compounds displayed significant antibacterial activity against all Gram-positive bacteria tested (Table 1, Entry 1 and 2), but were in accordance with the results of Krejci et al. [28] inactive against the Gram-negative ones. Furthermore, the authors proved on cultures of *S. aureus* that both compounds are bacteriostatic as well as bactericidal.

**Figure 6:**
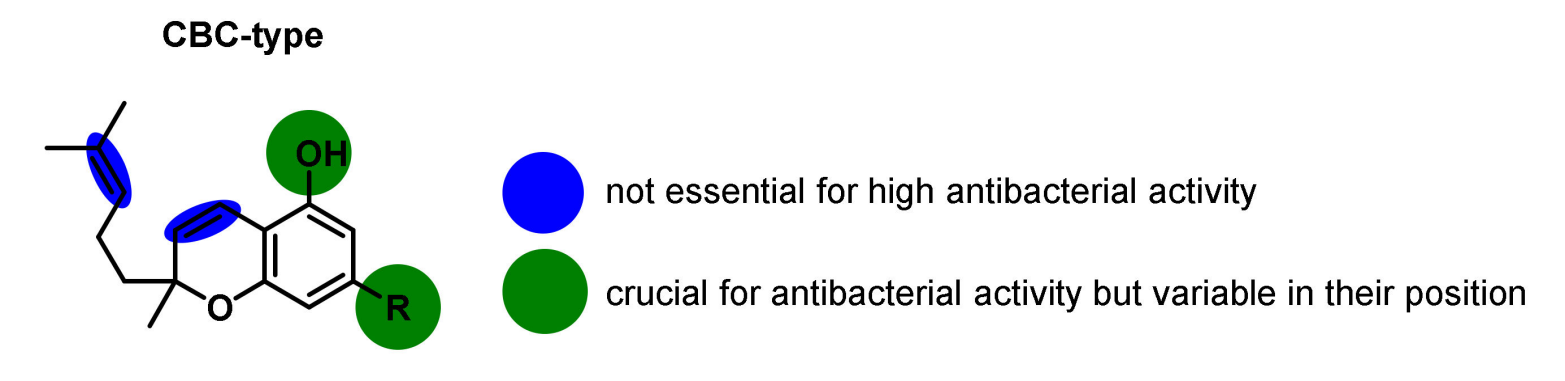
Illustration of the structure-activity relationships of CBC-type cannabinoids on the antibacterial activity.

In 1981, Turner and ElSohly published a report on the in vitro evaluation of antimicrobial activity of CBC-type cannabinoids against different Gram-positive bacteria and fungi including an in vivo study in rats investigating the anti-inflammatory activities in an carrageenan-induced rat paw edema assay [25]. In 1982, the same research group published an expanded study on the antibacterial and antimycotic activities of further CBC-type and CBG-type cannabinoid derivatives [29]. The results of both studies are summarized in Table 2. In this study, CBC (**19**) showed the highest activity of all tested CBC-type cannabinoids against *S. aureus* and *B. subtilis* with a MIC of 1.56 µg/mL and 0.39 µg/mL, respectively, even superior compared to Streptomycin (Table 2, Entry 1). Interestingly, *iso*CBC (**33**) (Table 2, Entry 2), bearing the lipophilic side chain and one hydroxyl group in exchanged positions displayed similarly potent activity against B. subtilis with a MIC of 0.78 µg/mL. The truncation of the lipophilic side chain in both *iso*CBC- and CBC-type cannabinoids led to a clear 2–8-fold drop in antibacterial activity (Table 2, Entries 3, 4 and 5), with the exception of *iso*CBC-C_1_ (**35**) exhibiting a MIC of 0.78 µg/mL against *B. subtilis* (Table 2, Entry 6). Moreover, the double bonds in the GPP-derived side chain seem to be less important for high activity as the reduced derivative **38** (Table 2, Entry 7) showed comparable antibacterial activity as CBC (**19**). Interestingly, a moderate antimycobacterial activity *Mycobacterium smegmatis* was observed for some of the compounds with best activity for CBC-C1 (**37**) and **38** exhibiting MIC values of 3.12 µg/mL comparable to Streptomycin (Table 2, Entry 4). The structure–activity relationships for the antibacterial activity of CBC-type cannabinoids can be concluded as illustrated in Figure 6. The antimycotic activity of the compounds was less pronounced. Only CBC-C_1_ (**37**) displayed a moderate antimycotic activity against *Sachcheromyces cerevisiae* with a MIC of 6.25 µg/mL, clearly less active compared to Amphotericin B. The activity against *Trichophyton mentagrophytes*, a pathogenic fungus, was comparable to Amphotericin B with 2 two-fold higher MIC of 6.25 µg/mL.

The authors also demonstrated that all tested CBC-type cannabinoids in this study reveal a higher anti-inflammatory activity compared to phenylbutazone or aspirin [25].

The CBG-type derivatives *iso*CBG-C_1_ (**39**) and CBG-C_1_ (**40**) displayed comparably strong antibacterial activity with MIC values in the range of 1.56–6.25 µg/mL [29]. According to the trends observed for the CBC-type derivatives, the reduction of the double bond in the GPP-derived side chain was well tolerated, as derivative **41** showed similar antibacterial activity against *S. aureus* and *B. subtilis* and good activity against *M. smegmatis* with a MIC of 3.12 µg/mL. Again, the antimycotic activities of the CBG-type compounds were less pronounced with only moderate activity. CBE (**14**) showed no noteworthy antibacterial or antimycotic activity [29].

**Figure 7:**
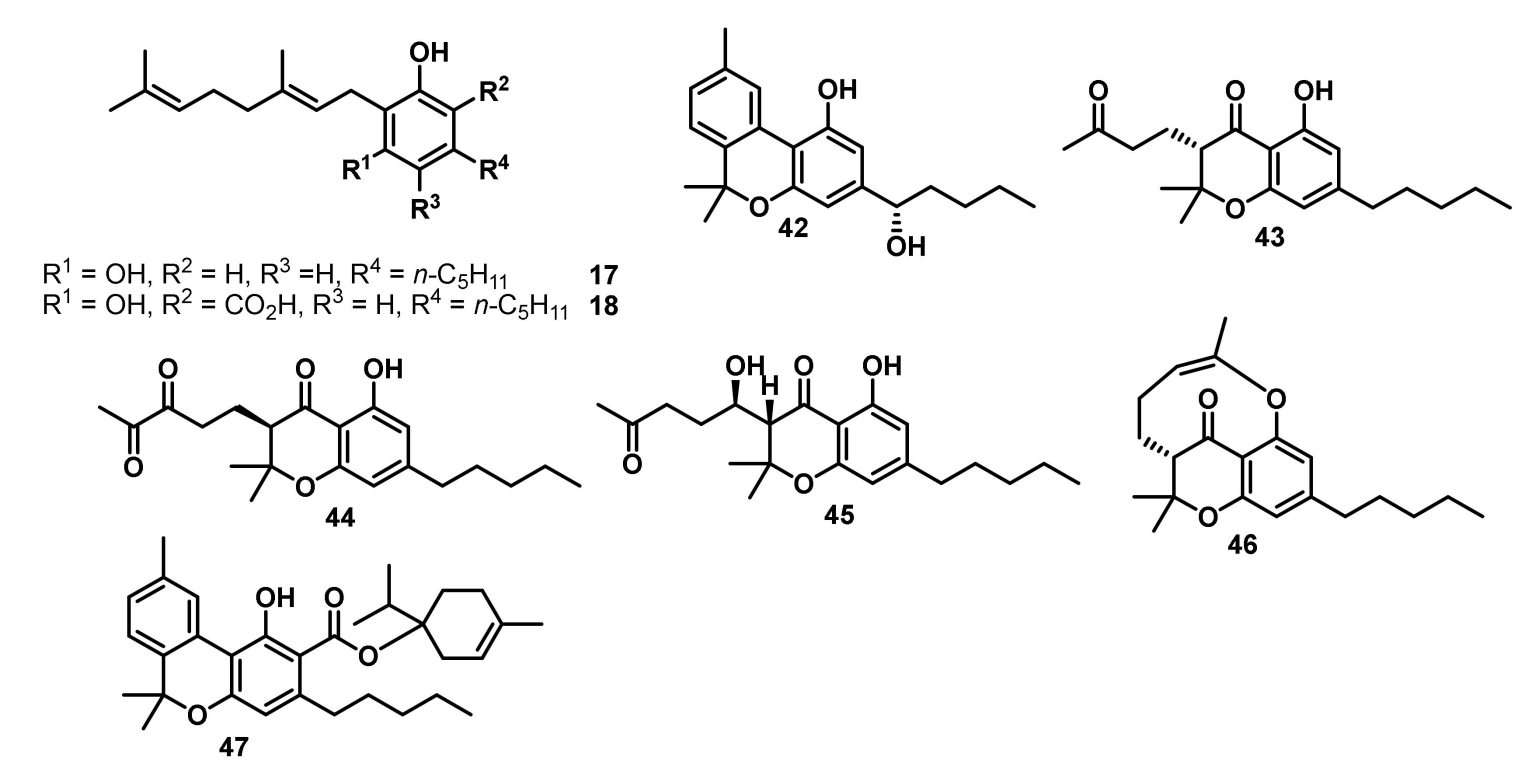
Structure of minor cannabinoids isolated from high potency Cannabis sativa by ElSohly, Ross, and co-workers [136,137,138,139].

Between 2008 and 2015, a consortium of researchers around *Ross* and *ElSohly* published a series of studies on the isolation and antimicrobial evaluation of novel minor cannabinoids from high potency variants of *Cannabis sativa* [136,137,138,139,140]. In their studies, CBG (**17**) (Figure 7) exhibited selective moderate antimicrobial activity against *Mycobacterium intracellulare* with an IC_50_ value of 15.0 μg/mL [137]. Furthermore, CBGA (**18)** displayed moderate antileishmanial activity with an IC_50_ value of 12.0 μg/mL against *Leishmania donovani*, while both compounds did not show any cytotoxic activity on African green monkey kidney fibroblast [137]. Compound **42** (Figure 7), a cannabinol derivative bearing an additional hydroxy function in the lipophilic side chain, showed moderate anti-MRSA (IC_50_ = 10.0 µg/mL), moderate antileishmanial (IC_50_ = 14.0 µg/mL), and reasonable antiprotozoal activity against *Plasmodium falciparum* (D6 clone) and *P. falciparum* (W2 clone) with IC_50_ values of 3.4 and 2.3 µg/mL, respectively [138]. The isolated cannabichromanone derivatives **43** and **44**, both differing in their oxidation pattern, displayed antiprotozoal activity against *P. falciparum* (D6 and W2 clone) with IC_50_ values of 3.7 and 3.8 µg/mL, and 4.7 and 3.4 µg/mL, respectively [139]. Meanwhile, the cannabichromanone derivatives **45** and **46** isolated in the same study showed no antiprotozoal activity. However, all of these cannabichromanone derivatives **43**–**46** showed moderate antileishmanial activity with IC_50_ values of 14.0, 14.0, 12.5, and 9.0 µg/mL, respectively [139]. Compound **47**, a prenylated ester of cannabinol, showed moderate antimycotic activity against *C. albicans* ATCC 90028, with an IC_50_ value of 8.5 µg/mL, and good antiprotozoal activity against *P. falciparum* (D6 clone) and *P. falciparum* (W2 clone), with IC_50_ values of 2.7 and 2.4 µg/mL, respectively. A CB1R binding assay indicated that **47** is non-psychotropic [136].

Compound **52** (Table 3, Entry 5), a hydroxylated derivative of CBNA, exhibited good antimycotic activity against *Candida albicans* (IC_50_ = 4.6 µM) as well as low antimycobacterial activity against *M. intracellulare* (IC_50_ 30.6 µM) [140]. The CBG derivative **49** (Table 3, Entry 2) and CBG derivative **51** (Table 3, Entry 4) possessed moderate anti-MRSA activity with IC_50_ values of 24.4 and 6.7 µM, respectively. Furthermore, both compounds displayed additional low antileishmanial activity against *L. donovani*. A good anti-MRSA activity with an IC_50_ value of 3.5 µM was observed also for the hydroxylated CBN derivative **53** (Table 3, Entry 6) as well [140]. The CBC-type derivative **48** (Table 3, Entry 1) and CBG derivative **50** (Table 3, Entry 3) showed moderate antiprotozoal activity against *P. falciparum* (D6 and W2 clone), while **50** additionally displayed a moderate antileishmanial activity with an IC_50_ value of 10.7 µM [140]. In these studies, rather, single values of inconsistently screened different antimicrobial activities were reported, thus, clear structure-activity relationships are hard to conclude.

In 2008, Appendino et al. [24] reported a more focused structure–activity relationship study on the anti-staphylococcal activity of different naturally occurring cannabinoids and synthetic derivatives against a broad range of various multi-drug resistant strains of *S. aureus,* including EMRSA-15, one of the main epidemic methicillin-resistant strains [141], SA-1199B, a multidrug-resistant strain that possesses a gyrase mutation, and in addition overexpresses the NorA efflux mechanism [142], RN-4220, a macrolide-resistant strain [143], XU212, and a tetracycline-resistant strain overexpressing the TetK efflux pump [144], as summarized in Table 4.

**Figure 8:**
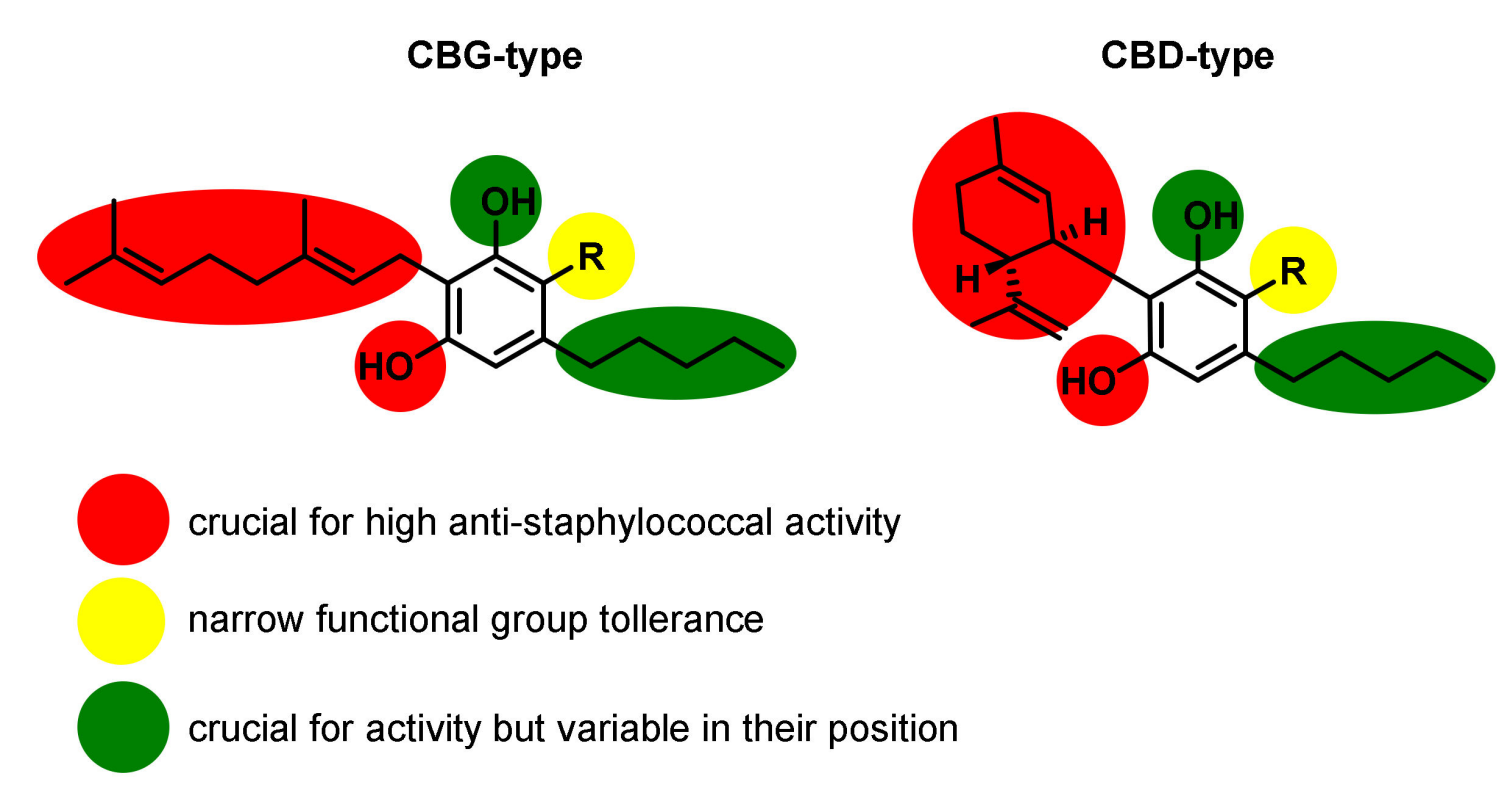
Illustration of the structure–activity relationships of cannabinoids of the CBG- and CBD-type on the anti-staphylococcal activity.

In their studies, all major cannabinoids Δ^9^-THC (**1**) (Table 4, Entry 10), CBD (**10**) (Table 4, Entry 1), CBG (**17**) (Table 4, Entry 5), CBC (**19**) (Table 4, Entry 4), and CBN (**7**) (Table 4, Entry 12) obtained by isolation from *Cannabis sativa* display potent anti-staphylococcal activity against all tested strains, with MIC values in the range of 0.5–2.0 µg/mL in most cases, even superior, compared to the reference antibiotics norfloxacin, erythromycin, tetracycline, and oxacillin. Given their non-psychotropic activity profiles, the authors selected CBG (**17**) and CBD (**10**), and synthetically generated several derivatives for further structure–activity studies, which are illustrated in Figure 8. The structural exploration revealed that in both cannabinoid types, the phenolic hydroxyl moieties are crucial for activity as mono- or di methylation, as mono-or deacetylation led to complete loss of anti-staphylococcal activity (Table 4, Entries 3, 7, 8, and 9). In light of the potent activity of the monophenols Δ^9^-THC (**1**) (Table 4, Entry 10), CBC (**19**) (Table 4, Entry 4), and CBN (**7**) (Table 4, Entry 12), the complete loss of activity for the mono methylation is not fully consistent and indicates a more complex SAR picture.

The carboxylic acid precursors CBGA (**18**) (Table 4, Entry 6) and CBDA (**11**) (Table 4, Entry 2) similar to Δ^9^-THC (**2**) (Table 4, Entry 11) retained their potent activity; however, esterification of the carboxylic acid moieties was detrimental for the activity. Thus, compounds **57, 58**, **62,** and **63** showed no noteworthy activity (Table 4, Entries 3, 8 and 9). Additionally, bigger substituents in the same position, such as in the prenylated CBD-derivative **67,** were also not tolerated and led to complete loss of activity. While the biosynthetic cannabinoid precursor olivetol **68** showed very low activity (Table 4, Entry 17), no activity at all was observed for resorcinol **69** missing the lipophilic side chain (Table 4, Entry 18). Thus, indicating that both the lipophilic C5 side chain as well as GPP-derived lipophilic side chain are crucial for high activity. In addition, the substantially decreased activity of camagerol (**66**), a dehydroxylated CBG-derivative revealed that the overall polarity in the GPP-derived lipophilic side chain is important for high activity as well. Interestingly, the synthetic isomers of CBD (Compound **64**, Table 4, Entry 13) and CBG (Compound **65**, Table 4, Entry 14), bearing the lipophilic side chain and one hydroxyl group in exchanged positions displayed potent activity comparable to their corresponding natural counterparts indicating that both moieties, although crucial for activity, can vary in their position at the phenolic core. A trend that has been observed for CBG-C_1_-type and CBC-type cannabinoid derivatives earlier [29], suggesting that they may rather serve as lipid affinity modulators influencing water solubility and cellular bioavailability [24].

Recently, a very comprehensive study on the antimicrobial, antibiofilm and anti-persister cell activities of phytocannabinoids from *Cannabis sativa* against MRSA was published by Brown and co-workers comprising in vitro and in vivo investigations of 18 commercially available cannabinoids, pre-cannabinoids and synthetic isomers [23]. The results of this study are summarized in Table 5.

Seven of the tested cannabinoids showed potent antibacterial activity against MRSA USA300, a highly virulent and prevalent pathogen, including Δ^9^-THC (**1**), Δ^8^-THC (**5**), CBN (**7**), CBD (**10**), CBG (**17**), CBCA (**20**), and exo-THC (**22**) (Table 5, Entries 1, 5–7, 11, 14 and 16), in accordance with previously published data [24,25,26,29]. In contrast to the study by Appendino et al. [24], in most cases, with the exception for CBCA (**20**) (Table 5, Entry 14), the authors observed a moderate loss of activity for the corresponding carboxylic acid bearing pre-cannabinoids. Thus, Δ^9^-THCA (**2**), CBDA (**8**) and CBGA (**18**) (Table 5, Entries 2, 8 and 12) were 2–8-fold less active than their decarboxylated congeners. Furthermore, the cannabinoids Δ^9^-THCV (**3**) and CBDV (**12**) (Table 5, Entries 3 and 9), bearing a truncated *n*-propyl lipophilic side chain were 2- and 4-fold less active compared to their congeners Δ^9^-THC (**1**) and CBD (**10**), respectively, bearing the *n*-pentyl side chain. The negative impact of side chain truncation and carboxylic acid substitution on the anti-staphylococcal activity was additive detrimental as both Δ^9^-THCVA (**4**) and CBDVA (**13**) (Table 5, Entries 4 and 10) exhibited an additional 4-fold decrease in anti-staphylococcal activity compared to Δ^9^-THCV (**3**) and CBDV (**12**), respectively. Noteworthy, both major human metabolites of Δ^9^-THC (**1**), 11-hydroxy-Δ^9^-THC (11-HO-Δ^9^-THC, **26**) and nor-9-carboxy-Δ^9^-THC (11-nor-9CTHC, **27**) (Table 5, Entries 17 and 18) as well as CBL (21) Table 5, Entry 15) were inactive at the highest screen concentration.

Two major features strongly contributing to the high virulence and strong persistence of MRSA in both human host organism and environment are its high notion to form biofilms on necrotic tissue and medical devices [13,14] and its ability to form non growing, dormant persister subpopulations [145,146,147,148,149]. Both making the pathogen less susceptible for host immune factors displaying high levels of tolerance to antibiotics, such as gentamicin, ciprofloxacin, and vancomycin [145,146,147,148,149], and can led to severe chronic infections [150,151,152].

Recently, antibiofilm activity of ethanolic extracts of *Cannabis sativa,* likely containing phytocannabinoids, has been reported by Frasinetti et al. [121]. However, the study of Brown and co-workers for the first time allowed a direct attribution of antibiofilm activity to the tested cannabinoid derivatives [23]. As summarized in Table 5, the antibiofilm activity of all tested cannabinoids against MRSA USA300 strain correlated well with their specific anti-staphylococcal activity. Thus, the major cannabinoids, such as Δ^8^-THC (**5**), CBGA (**18**), CBG (**17**), and exo-THC (**22**) completely suppressed biofilm formation at concentration of 2 µg/mL (Table 5, Entries 5, 11, 12 and 16), CBG (**17**) displaying the most potent antibiofilm activity with an IC_50_ value of 0.5 µg/mL [23]. Remarkably, in addition, CBG (**17**) was demonstrated to eradicate preformed biofilms of MRSA USA300 at concentrations of 4 µg/mL [23]. Brown and co-workers also screened for the ability of the cannabinoids to kill persister cells (Table 5). The anti-persister activity correlated with the activity against actively dividing cells and CBG (**17**) showed the most active cannabinoid killing persisters in concentration-dependent manner starting at 5 µg/mL, again. Impressively, CBG (**17**) was able to completely eradicate a population of ~108 CFU/mL MRSA persisters below detection thresholds within 30 min of treatment [23].

In order to elucidate the molecular target of CBG (**17**), Brown and co-workers repeatedly challenged MRSA USA300 with various lethal concentrations ranging from 2–16-fold MIC or sequential subcultures in solution with sublethal concentration of CBG (**17**) to select for spontaneous resistance. However, no spontaneous resistant mutants were obtained, suggesting a low frequency of resistance of below 10^−10^ for MRSA [23]. A comprehensive chemical genomic analysis on the model bacterium *B. subtilis* at sublethal concentrations of CBG (**17**) than led to the identification of 41 transposon mutants, which revealed an enrichment of genes encoding for proteins that are localized at the cytoplasmic membrane, and genes encoding for proteins involved in processes taking place at the cytoplasmic membrane. Thus, indicating that CBG (**17**) acts via a disruption of the integrity of the cytoplasmic membrane, which was confirmed through treatment of MRSA cells with a membrane-potential sensitive fluorescent probe in the presence of CBG (**17**), leading to a dose-dependent increase of fluorescence occurring at MIC of CBG (**17**) [23]. Unfortunately, a hemolytic activity of CBG (**17**) on human erythrocytes was observed at 32 µg/mL, above its MIC of 2 µg/mL, but with a low therapeutic index of 16:1 [23].

However, a pharmacokinetic study of 120 mg/kg doses of CBG (**17**) in rats and mice have reported no signs of acute toxicity [153]. Brown and co-workers demonstrated the antibacterial efficacy of CBG (**17**) in vivo at 100 mg/kg doses in a murine systemic infection model, displaying a significant reduction of bacterial burden by a factor of 2.8 log_10_ in CFU compared to the control, while being well tolerated in mice [23].

Finally, Brown and co-workers discovered that the antibacterial activity of several cannabinoids could be significantly increased against Gram-negative pathogens, which have been reported to be less susceptible towards cannabinoids [26,140] in the presence of sublethal concentrations of polymyxins perturbating the outer membrane of Gram-negative bacteria. The authors could show, that CBG (**17**), which was inactive against *E. coli* (>128 µg/mL), was active in the presence of a sublethal concentration of 0.062 µg/mL of polymyxin B with a MIC of 1 µg/mL.

This observation uncovered the hidden broad-spectrum antibiotic activity within this synergistically acting polymyxin B adjuvant combination therapy approach, and allowed Brown and co-workers to demonstrate the potent efficacy of CBG (**17**) against clinical isolates of Gram-negative priority pathogens such as *Acinetobacter baumannii, E. coli*, *Klebsiella pneumoniae,* and *Pseudomonas aeruginosa*.

## 5. Conclusions

Novel antimicrobial drugs are urgently needed to counteract the growing occurrence of bacterial resistance towards market antibiotics. Phytocannabinoids, including Δ^9^-THC-type, Δ^8^-THC-type, CBN-type, CBD-type, CBC-type, and CBG-type derivatives have been demonstrated to display potent antibacterial activity against Gram-positive pathogens, in particular against highly virulent and prevalent MRSA strains, which are the leading cause of both healthcare and community-associated infections [23,24,25,26,29]. Due to their non-psychotropic and non-sedative pharmacologic properties, CBD-type, CBC-type, and in particular CBG-type cannabinoids are the most promising candidates for further investigations. Although, the carboxylic acid bearing pre-cannabinoids, such as Δ^9^-THCA (**2**) or Δ^8^-THCA (**6**) do not show psychotropic effects either, their intrinsic instability through sun light or heat mediated decarboxylation or aromatization in the presence of oxygen leading to psychotropic Δ^9^-THC-type, Δ^8^-THC-type or CBND-type cannabinoids, makes them less attractive for further development. The recently demonstrated potent antibacterial in vivo efficacy [23], which previously published reports on the inactivation of CBG (**17**) by serum [135] proved to be wrong, the discovery of additional potent antibiofilm and anti-persister cell activities of CBG (**17**) against MRSA [23], as well as the synergistic activity of CBG (**17**) in the presence of sublethal concentrations of polymyxins against Gram-negative priority pathogens, such as *A. baumannii, E. coli*, *K. pneumoniae,* and *P. aeruginosa,* further support the role of CBG-type cannabinoids as lead candidates for the development of novel broad spectrum antibiotics.

However, considering the complex pharmacology [67,68,154] of CBG (**17**) acting within the endocannabinoids system as an inhibitor for the uptake of the endocannabinoid ligand anandamide (**24**) [155] and, furthermore, taking into account the crucial role of the endocannabinoid system within a multitude of essential physiological processes [66,70,73,156], such as embryo and child brain development [157,158], more studies on the pharmacological profile of CBG (**17**) are necessary to elucidate the poorly understood pharmacological relationships. For non-psychotropic natural cannabinoids, mostly mild, tolerable side-effects such as dizziness, dry mouth, gastrointestinal disorders, or tiredness have been reported; however, this might change drastically for derivatives and needs to be investigated [87,88]. For CBG (**17**), although hemolytic activity on human erythrocytes with a low therapeutic index have been an issue in vitro [23], preliminary pharmacokinetic results on in vivo show no acute toxicity and high tolerance in mice and rats [23,153,159].

Finally, it is important to emphasize that, owing to their ability to activate the reward system, cannabinoid consumption is potentially addictive, and long-term use might produce tolerance and dependence [87,88]. Nevertheless, considering relatively short administration periods of antimicrobial drugs, this is probably less of an issue.

While CBG (**17**) possess several desirable physiochemical properties as a medicinal chemistry lead in terms of molecular weight, number of hydrogen donors and acceptors, number of rings and rotatable bonds, it is suffering from its high lipophilicity with a calculated logP of 6.74 and an associated poor water solubility, [23] which need to be addressed in medicinal chemistry campaigns. Furthermore, the accumulation of therapeutic cannabinoids in adipocytes of fatty tissue [160,161] due to their highly lipophilic nature need to be investigated to exclude negative long-term effects. Thereby, the easy access through isolation from high content types of *Cannabis sativa* and easy synthesis of CBG (**17**) from readily available starting materials [162] are a huge benefit, which will boost the chemical exploration of CBG (**17**) as antibacterial lead candidate.

Cannabinoids can be administered through vaporization, intravenous injection, sublingual mouth spray, or oral application, for the latter, however, appropriate stomach-resistant galenic formulations have to be considered to avoid known gastric degradation [161], leading to lowered plasma levels [153].

The most promising and obvious application of CBG (**17**) based antibiotics might be in topical applications against MRSA caused skin infections as the usage of cannabinoids in medical salve has already been successfully demonstrated by Krejci [101] in 1959, and mupirocin, the standard-of-care antibiotic in this indication, suffers from resistances [163,164].

Beyond all irrational hype on cannabis products, cannabinoids are truly promising antimicrobial drugs and I am convinced that they will find their way into a novel medical application as antibacterial treatment of infections. With regard to that, more research to address structural and pharmacokinetic shortcomings is needed.

## Figures and Tables

**Table 1:** Overview on antibacterial activity of Δ^9^-THC (**1**) and CBD (**10**) against different strains of Gram-positive and Gram-negative bacteria [26].

Entry	Compounds	Antibacterial Activity MIC [µg/mL]						
		*S. aureus ^a^*	*S. pyogenes*	*S. milleri*	*S. faecalis*	*E. coli*	*S. typhi*	*P. vulgaris*
1	Δ^9^-THC (**1**)	2–5 ^a^	5	2	5	>100	>100	>100
2	CBD (**10**)	1–5 ^a^	2	1	5	>100	>100	>100
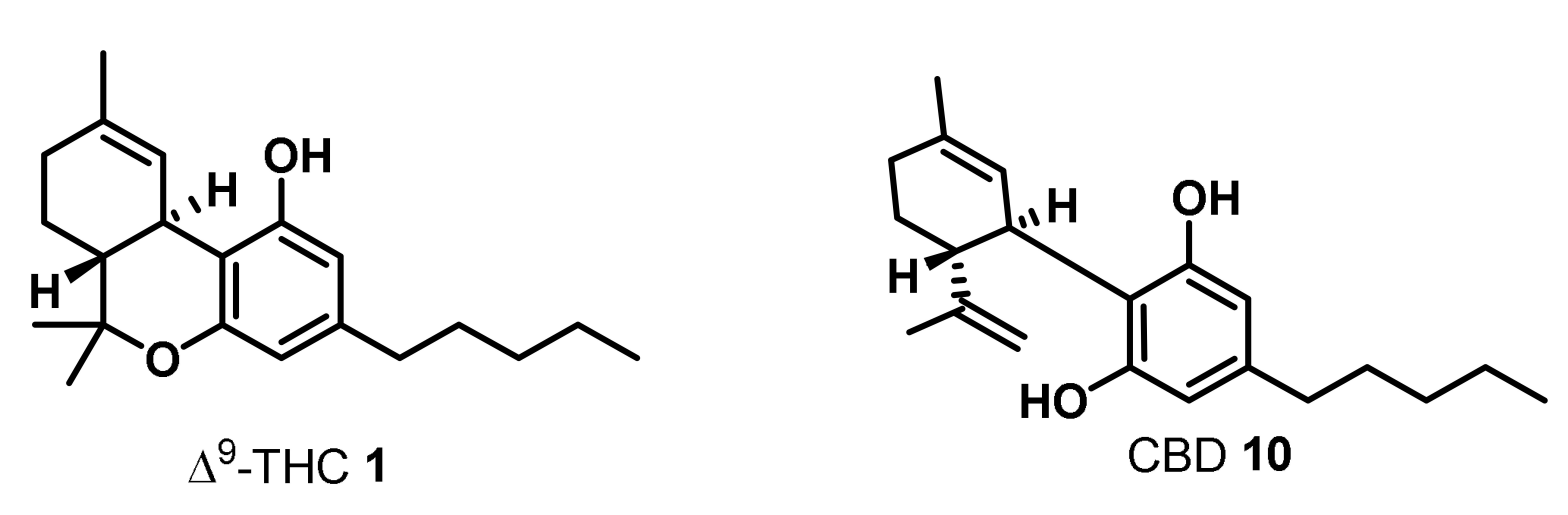								

^a^ 4 strains were tested.

**Table 2:** Overview on antibacterial and antimycotic activity of natural cannabinoids against different strains of bacteria and fungi [25,29].

Entry	Compounds	Antibacterial Activity MIC [µg/mL]			Antimycotic Activity MIC [µg/mL]		
		*S. aureus ^a^*	*B. subtilis ^a^*	*M. smegmatis ^a^*	*C. albicans ^b^*	*S. cerevisiae ^b^*	*T. mentagrophytes ^b^*
1	CBC (**19**)	1.56	0.39	12.5	nt	25	25
2	*iso*CBC (**33**)	nt	0.78	25.0	50	nt	nt
3	CBC-C_0_ (**36**)	12.5	6.25	12.5	50	25	25
4	CBC-C_1_ (**37**)	3.12	3.12	3.12	nt	6.25	6.25
5	*iso*CBC-C_0_ (**34**)	12.5	6.25	12.5	12.5	nt	nt
6	*iso*CBC-C_1_ (**35**)	nt	0.78	25.0	50.0	nt	nt
7	**38**	0.78	1.56	3.12	nt	12.5	50.0
8	*iso*CBG-C_1_ (**39**)	3.12	1.56	6.25	25.0	12.5	6.25
9	CBG-C_1_ (**40**)	1.56	1.56	6.25	25.0	6.25	6.25
10	**41**	1.56	0.78	3.12	12.5	6.25	25.0
11	CBE (**14**)	50	25	50	50.0	50.0	25.0
12	Streptomycin	6.25	3.12	1.56			
13	Amphotericin B				1.56	0.19	12.5
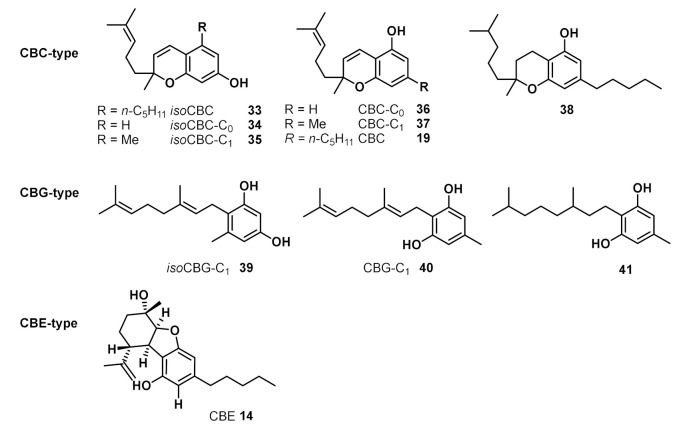							

^a^ MIC (Minimal Inhibitory Concentration) values after 24 h. ^b^ MIC values after 48 h. nt = not tested.

**Table 3:** Overview on antibacterial, antiplasmodial, and antimycotic activity of natural cannabinoids against different strains of bacteria, protozoa, and fungi [140]. (*C. krusei = Candida krusei*).

Entry	Compounds	Antibacterial Activity IC_50_ [µM]				Antimycotic Activity MIC IC_50_ [µM]		Antileishmanial Activity MIC IC_50_ [µM]	Antiprotozoal Activity IC_50_ [µM]	
		MRSA	*S. aureus*	*E. coli*	*M. intracellulare*	*C. albicans*	*C. krusei*	*L. donovani*	*P. falciparum*	
									*D6*	*W2*
1	48	nt	nt	nt	nt	nt	nt	40.3	7.2	4.0
2	49	24.4	29.6	na	na	60.5	60.5	57.5	na	na
3	**50**	53.4	na	na	na	na	nt	10.7	7.2	6.7
4	**51**	6.7	12.2	na	na	na	53.4	42.7	na	na
5	**52**	nt	nt	na	30.6	4.6	nt	nt	nt	nt
6	**53**	nt	3.5	54.0	na	na	54.0	nt	nt	nt
7	Ciprofloxacin	0.4	0.4	0.1	1.5					
8	Amphotericin B					0.3	0.7			
9	Pentamidine							3.8		
10	Chloroquine								0.1	0.5
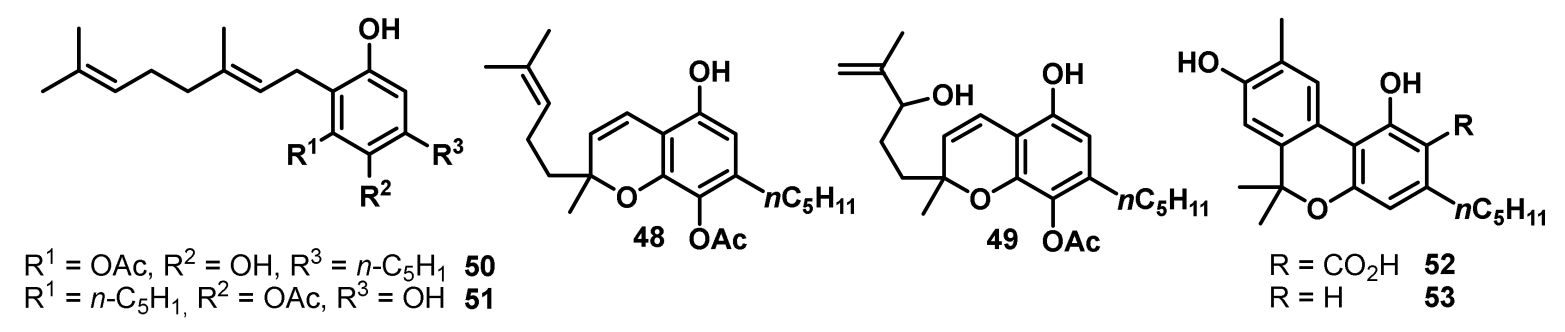										

na = no activity; nt = not tested.

**Table 4:** Overview on antibacterial activity of natural and synthetic cannabinoids and biosynthetic precursors against different strains of *Staphylococcus aureus* [24].

Entry	Compound	Antibacterial Activity MIC [µg/mL]					
		SA-1199B ^a^	RN-4220 ^b^	XU212 ^c^	ATCC25923 ^d^	EMRSA-15 ^e^	EMRSA-16 ^e^
1	CBD **10**	2	2	2	2	2	2
2	CBDA **11**	1	1	1	0.5	1	1
3	**54–58**	>100	>100	>100	>100	>100	>100
4	CBC **19**	2	2	1	2	2	2
5	CBG **17**	4	2	4	4	2	1
6	CBGA **18**	1	1	1	1	2	1
7	**59–61**	>100	>100	>100	>100	>100	>100
8	**62**	64	nt	64	nt	nt	nt
9	**63**	>100	>100	>100	>100	>100	>100
10	Δ^9^-THC **1**	8	4	8	4	8	4
11	Δ^9^-THCA **2**	2	1	1	1	2	0.5
12	CBN **7**	1	1	1	1	1	nt
13	**64**	1	1	1	1	1	1
14	**65**	2	1	0.5	1	2	nt
15	**66**	32	32	16	16	16	32
16	**67**	>100	>100	>100	>100	>100	>100
17	**68**	64	64	64	128	64	64
18	**69**	>100	>100	>100	>100	>100	>100
19	norfloxacin	32	1	4	1	0.5	128
20	erythromycin	0.25	64	>128	0.25	>128	>128
21	tetracycline	0.25	0.25	128	0.25	0.125	0.125
22	oxacillin	0.25	0.25	128	0.125	32	>128
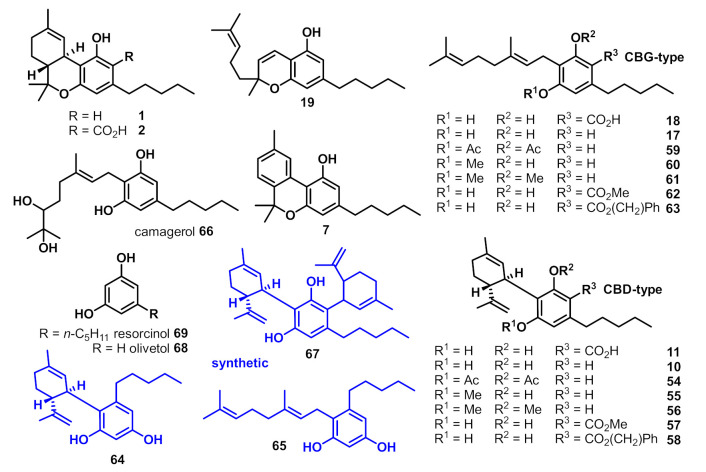							

^a^ SA1199B: multi-drug resistant *S. aureus* overexpressing NorA efflux pump. ^b^ RN-4220: macrolide resistant *S. aureus*. ^c^ XU212: tetracycline resistant S. aureus overexpressing TetK efflux pump. ^d^ ATCC25923: standard laboratory strain of *S. aureus*. ^e^ EMRSA-15 and EMRSA-16 (EMRSA = Epidemic meticillin-resistant Staphylococcus aureus): multi-drug and Methicillin-resistant *S. aureus* strains; nt = not tested.

**Table 5:** Overview on antibacterial, antibiofilm and anti-persister activity of natural and synthetic cannabinoids and biosynthetic precursors against methicillin-resistant *Staphylococcus aureus* (MRSA) USA300 [23].

Entry	Compound/Structure	Antibacterial Activity against MRSA USA300 ^a^ MIC [µg/mL]	Anti-Biofilm Activity [µg/mL] 100% Inhibition	Anti-Persister Cell Activity
1	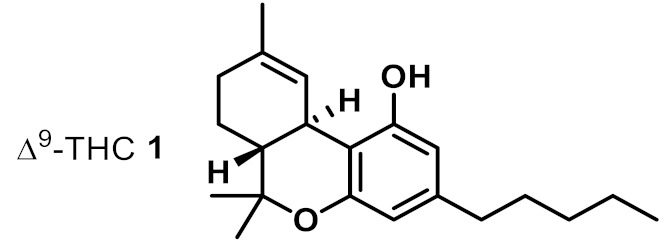	2	4	++
2	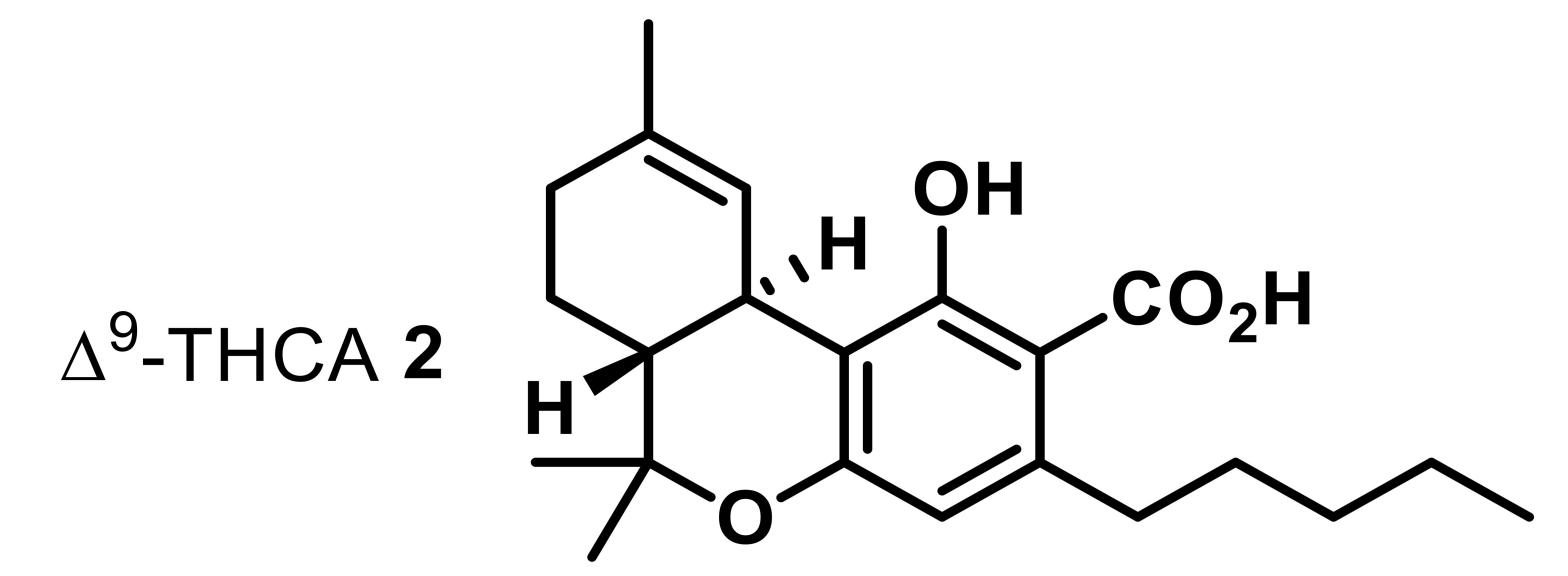	4	4	++
3	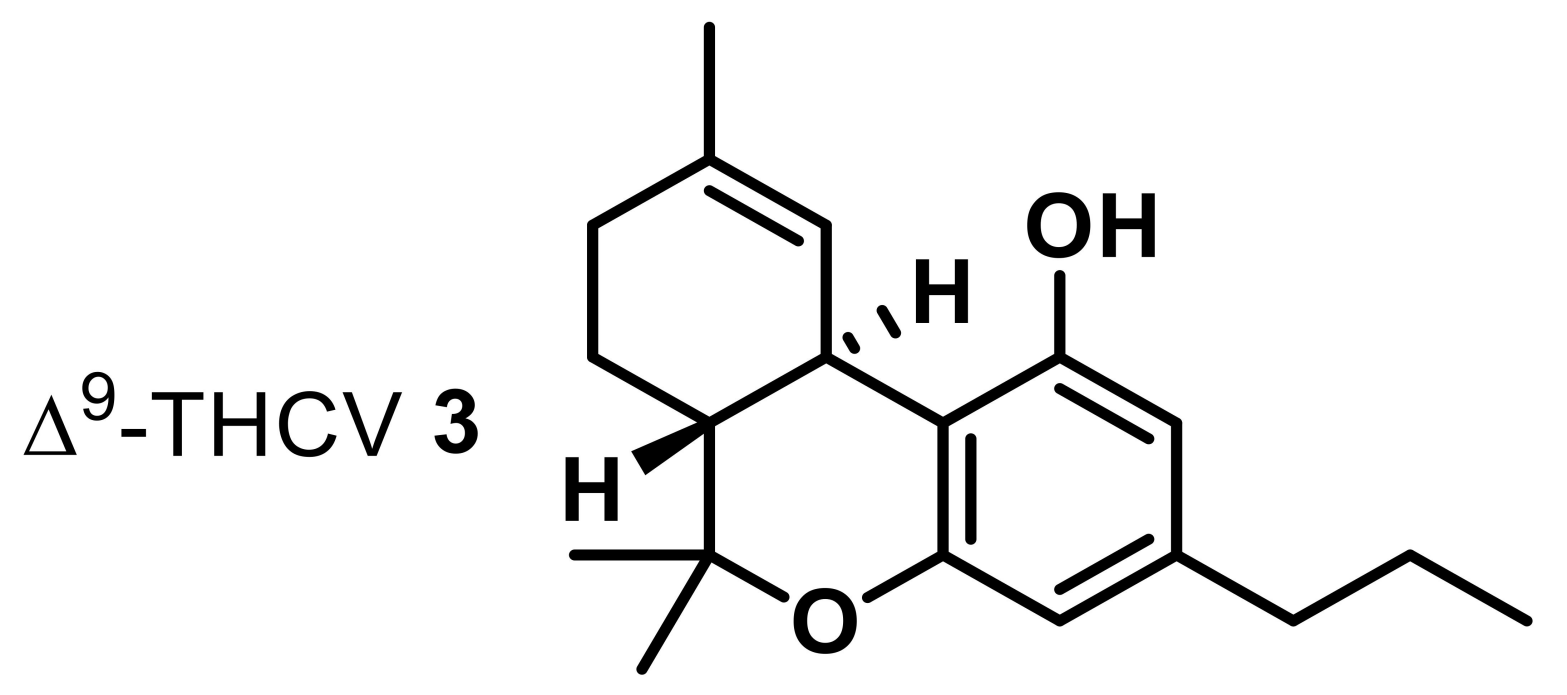	4	8	++
4	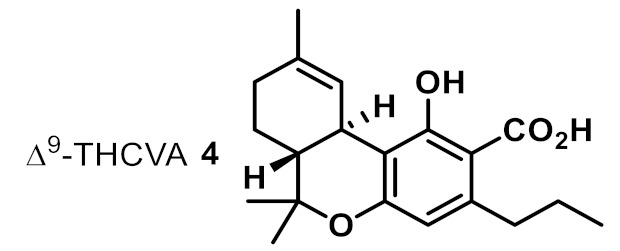	16	>16	na
5	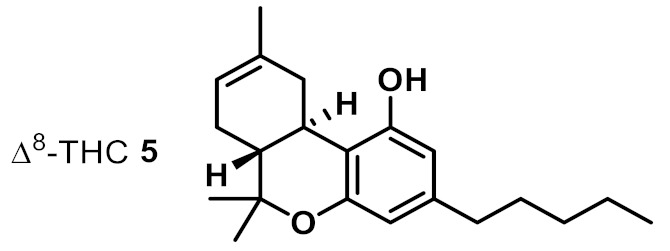	2	2	++
6	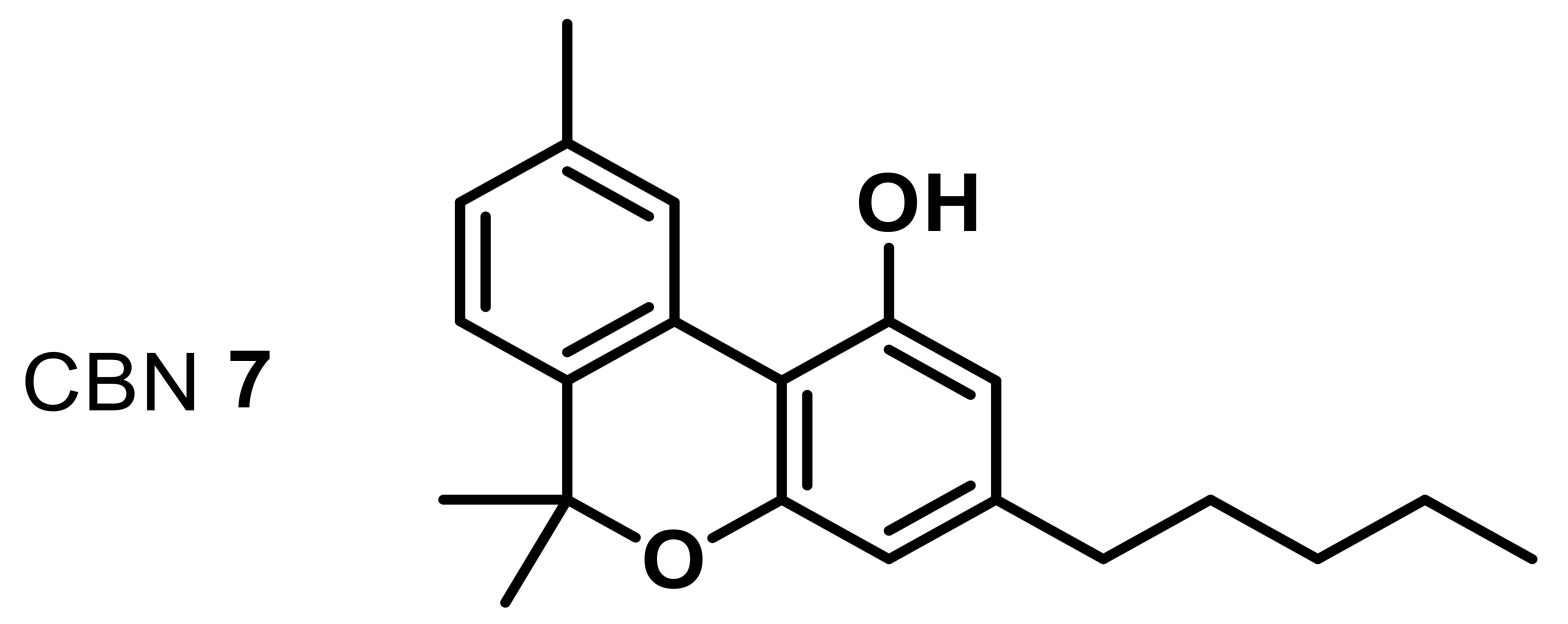	2	4	++
7	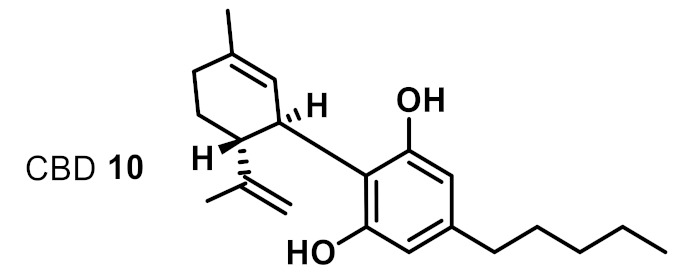	2	4	+++
8	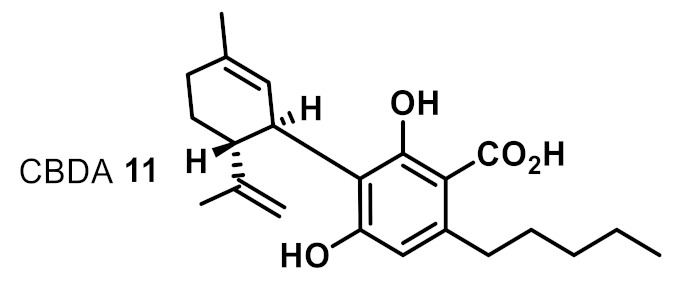	16	8	+
9	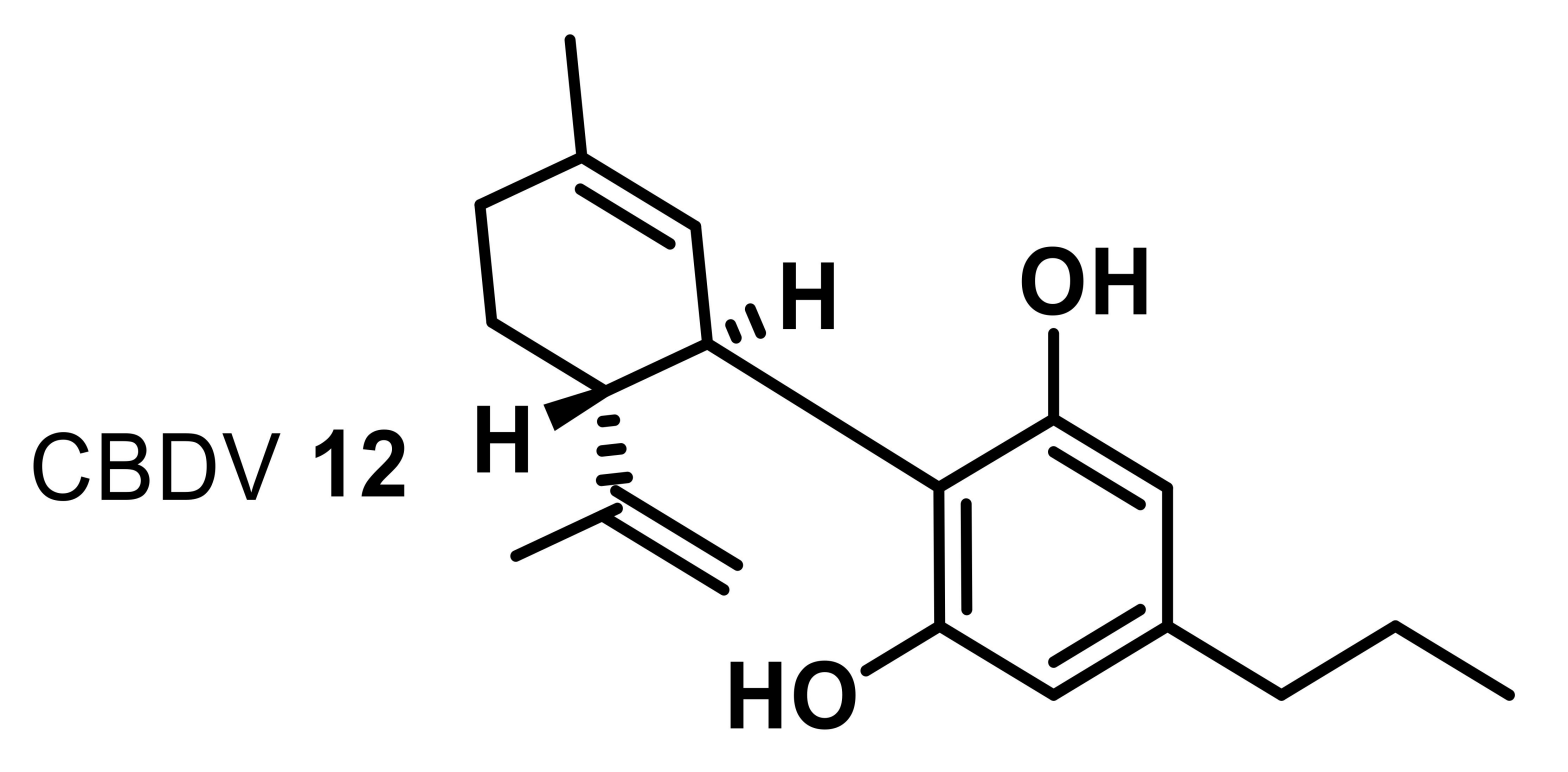	8	8	++
10	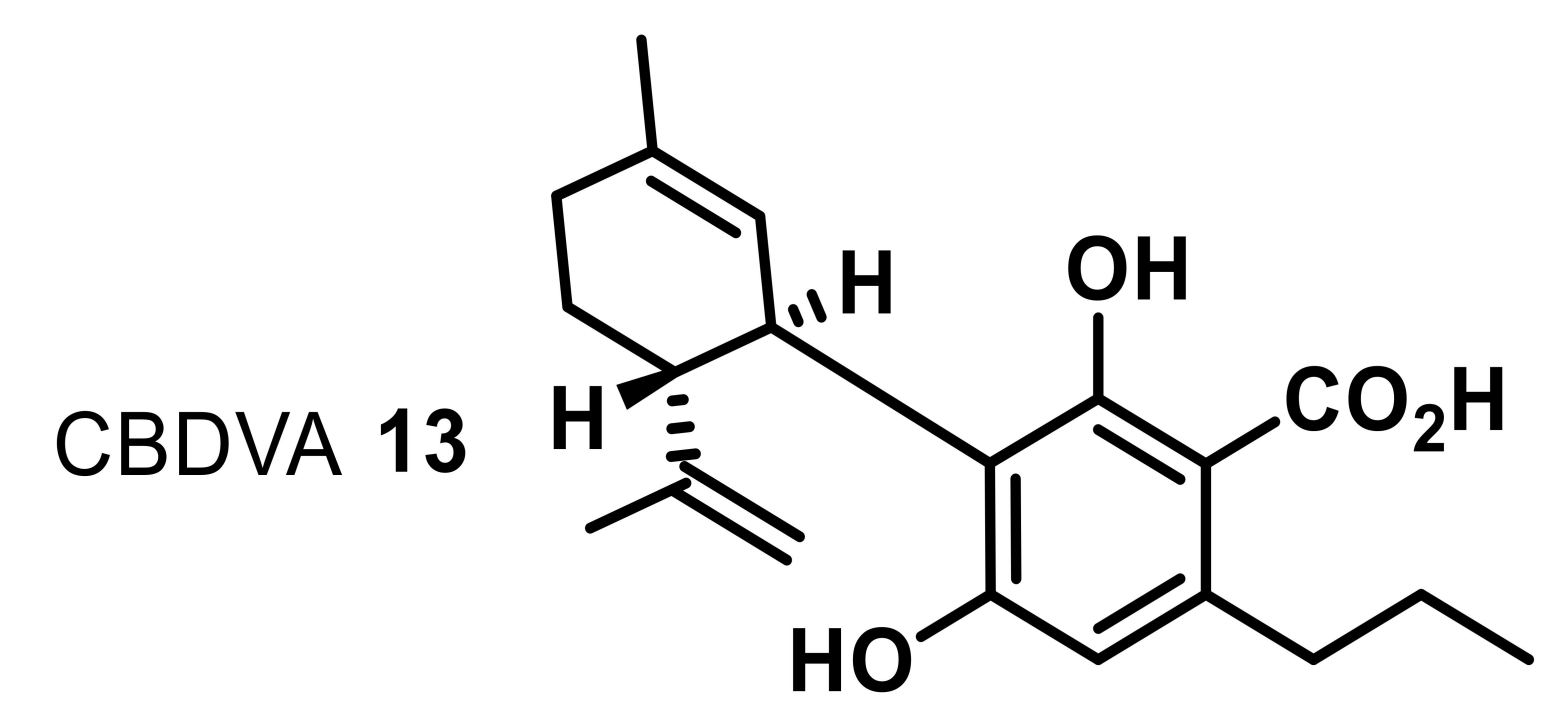	32	na	na
11	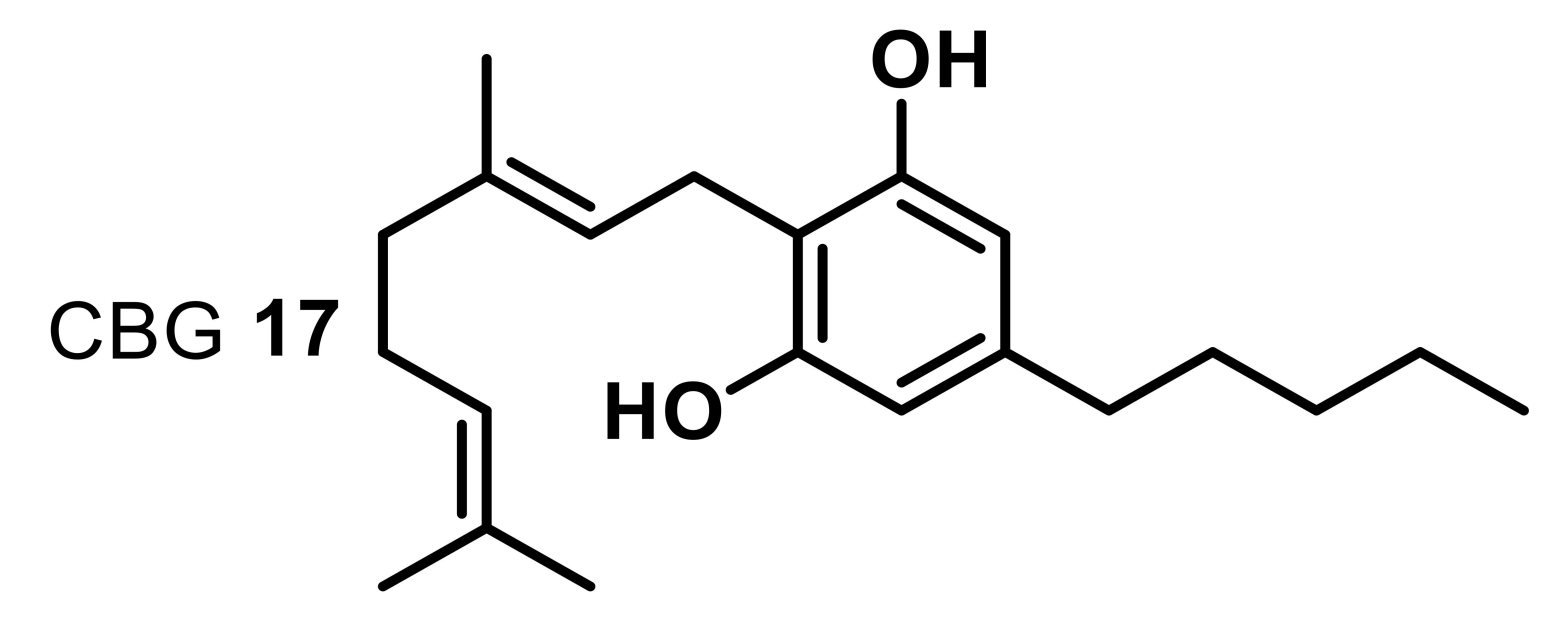	2	2	++++
12	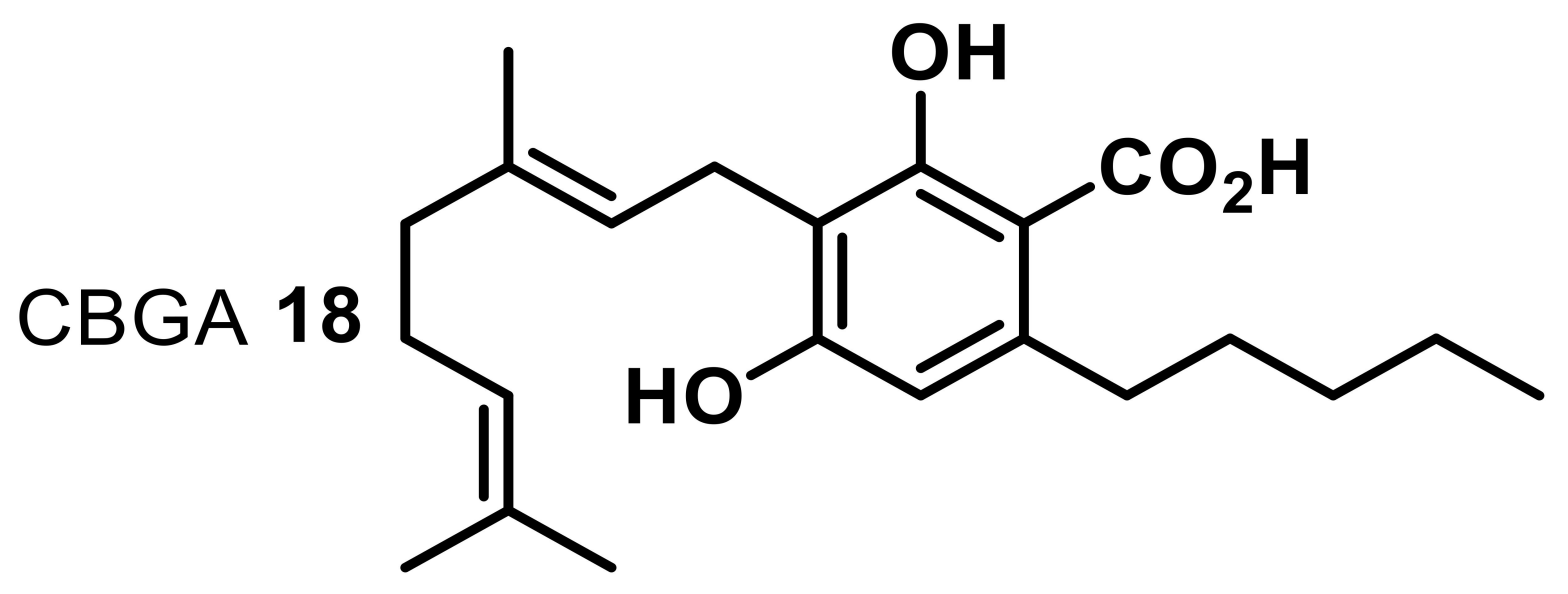	4	4	++++
13	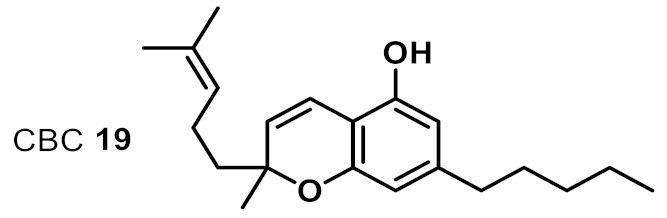	8	8	++
14	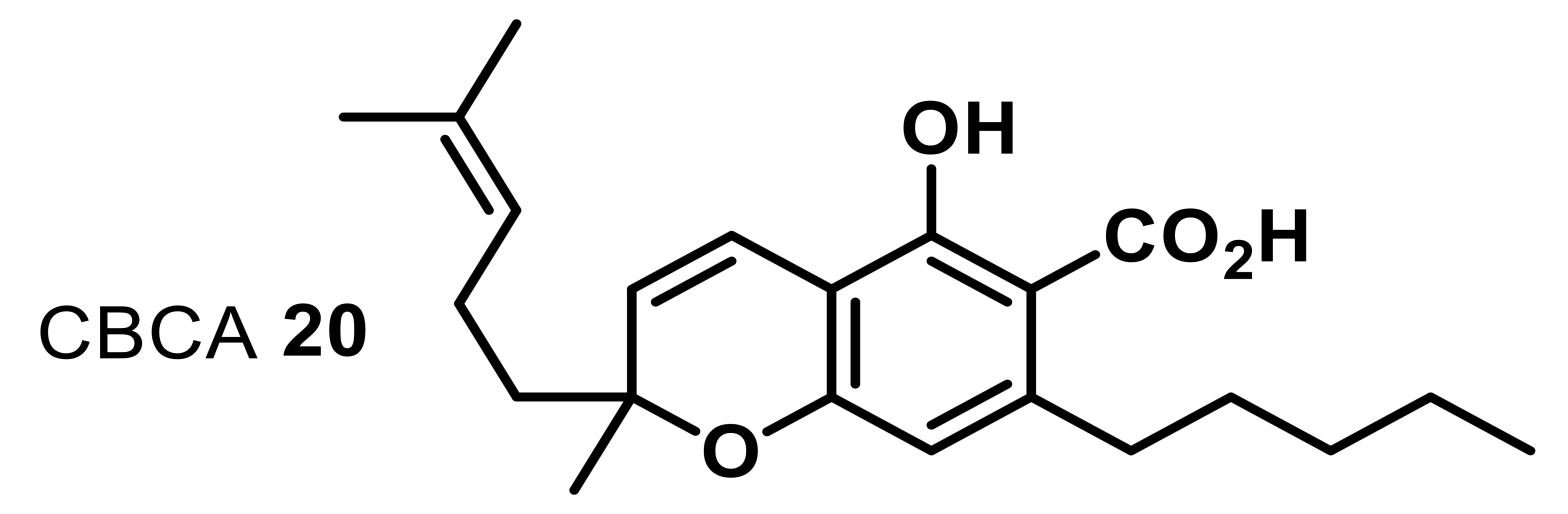	2	4	+++
15	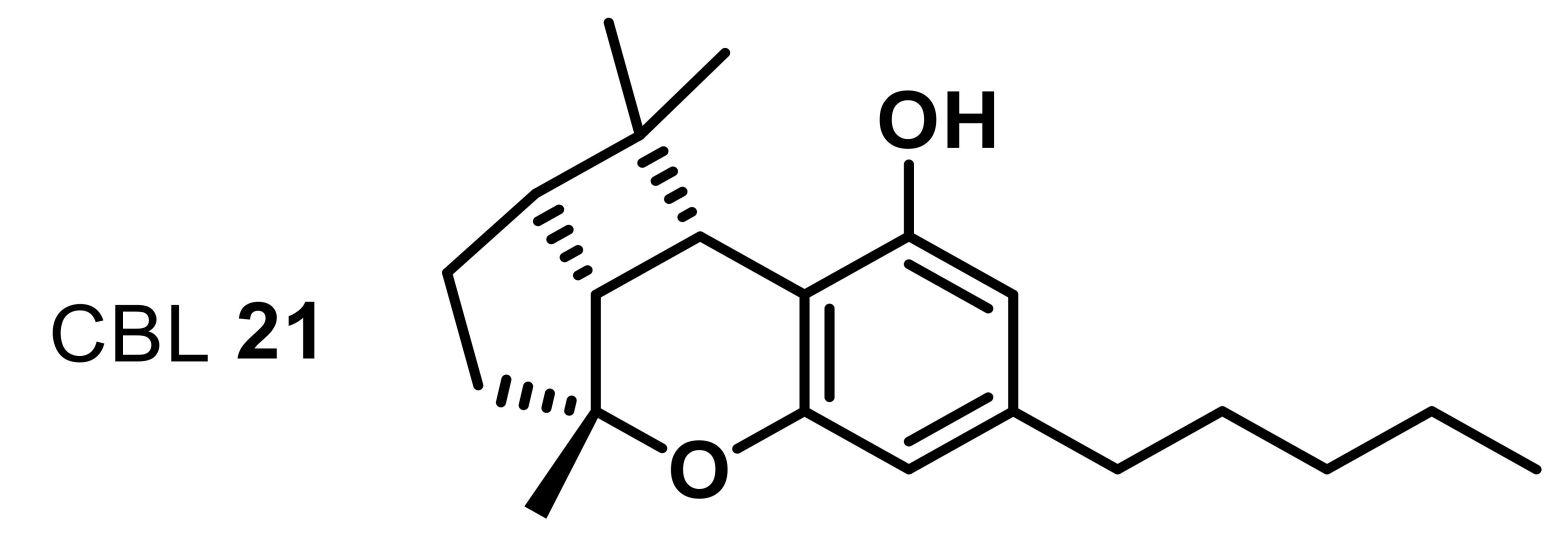	>32	na	na
16	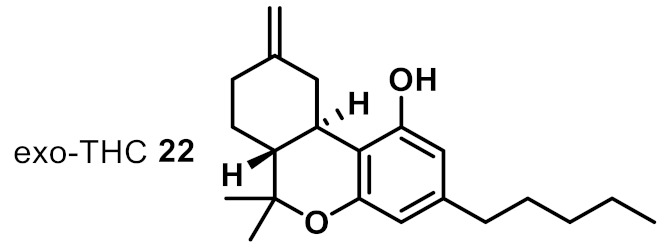	2	2	++
17	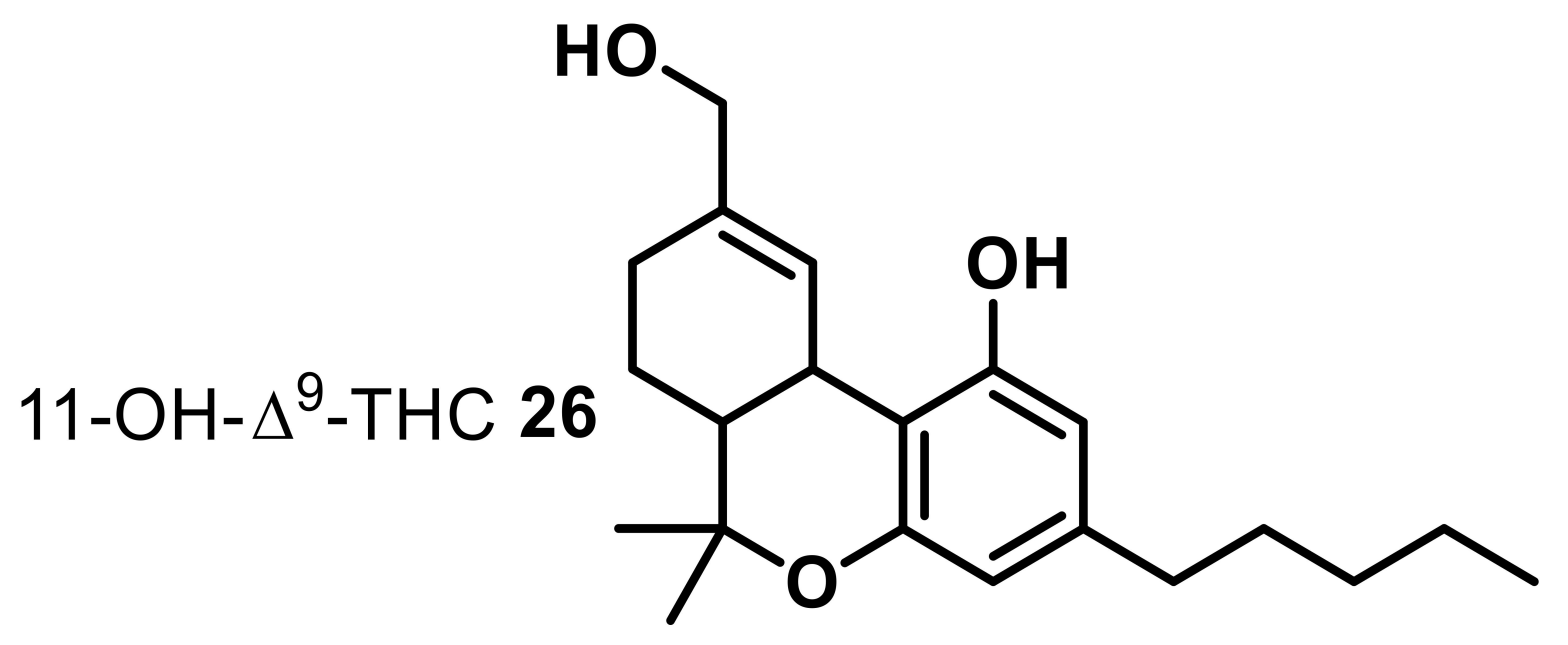	>32	~8	na
18	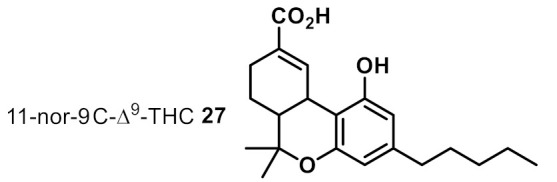	>32	na	na

^a^ MRSA USA300: highly virulent and prevalent community-associated multi-drug resistant *S. aureus.* na = no activity. Anti-persister cell actoivity: ++++ = very strong, +++ = strong, ++ = moderate, + = weak.
